# *Drosophila* Ninjurin A Induces Nonapoptotic Cell Death

**DOI:** 10.1371/journal.pone.0044567

**Published:** 2012-09-28

**Authors:** Sarah Broderick, Xiaoxi Wang, Nicholas Simms, Andrea Page-McCaw

**Affiliations:** 1 Department of Cell and Developmental Biology and Program in Developmental Biology, Vanderbilt Medical Center, Nashville, Tennessee, United States of America; 2 Department of Cancer Biology, Vanderbilt Medical Center, Nashville, Tennessee, United States of America; 3 Department of Biology, Rensselaer Polytechnic Institute, Troy, New York, United States of America; National Institutes of Health, United States of America

## Abstract

Ninjurins are conserved transmembrane proteins that are upregulated across species in response to injury and stress. Their biological functions are not understood, in part because there have been few *in vivo* studies of their function. We analyzed the expression and function of one of three *Drosophila* Ninjurins, NijA. We found that NijA protein is redistributed to the cell surface in larval immune tissues after septic injury and is upregulated by the Toll pathway. We generated a null mutant of *NijA*, which displayed no detectable phenotype. In ectopic expression studies, NijA induced cell death, as evidenced by cell loss and acridine orange staining. These dying cells did not display hallmarks of apoptotic cells including TUNEL staining and inhibition by p35, indicating that NijA induced nonapoptotic cell death. In cell culture, NijA also induced cell death, which appeared to be cell autonomous. These *in vivo* studies identify a new role for the Ninjurin family in inducing nonapoptotic cell death.

## Introduction

Ninjurins are a conserved family of transmembrane proteins first identified by upregulation in injured rat nerves [1]. There are two Ninjurin family members in mammals, Ninjurin1 and Ninjurin2 [2], and three in *Drosophila*, Ninjurin A, B, and C [3]. Ninjurins are small proteins of ∼16–27 kDa, with an N-terminal ectodomain and two predicted transmembrane domains near the C-terminal end. In humans, mice, and *Drosophila*, Ninjurin transcripts are upregulated upon injury, infection, or stress suggesting that not just their structure but also their function is conserved [1], [4], [5], [6], [7], [8], [9] (and see supplemental data in [10], [11], ).

Although little is known about the functions of Ninjurins, many studies have implicated them as adhesion molecules, either directly through homophilic binding on the cell surface [1], [13], [14], [15] or by regulating adhesion via their ectodomain [3]; yet these adhesion studies have been limited to cell culture models. *In vivo*, some data suggests that Ninjurin1 may promote hyaloid vasculature regression in mouse embryos, as neutralizing antibodies against Ninjurin1 delay this regression, although the relationship between Ninjurin1 and cell death *in vivo* is unclear from these studies [14]. To our knowledge, no Ninjurin mutants or knock-outs have been reported in any organism.

In this study, we show that Ninjurin A (NijA) protein responds to septic injury in a developmentally regulated manner, as whole-animal levels increase in adults but not in larvae. Rather, in larvae the protein distribution is altered in immune tissues after injury, and NijA protein levels can be elevated via the Tl immune signaling pathway, suggesting that NijA may function in the immune system. We generated several deletion mutants of *NijA* including a molecular null allele but no phenotype was observed in these animals. In a gain-of-function approach, however, we found that *NijA* induced cell death at a level comparable to the known apoptotic gene *hid*, yet the NijA-induced death does not have the hallmarks of apoptosis. From cell culture studies, we conclude that NijA is likely to induce cell death in a cell-autonomous manner, rather than as a nonautonomous signaling molecule.

## Results

### NijA distribution is regulated in larval immune tissues

**Figure 1:**
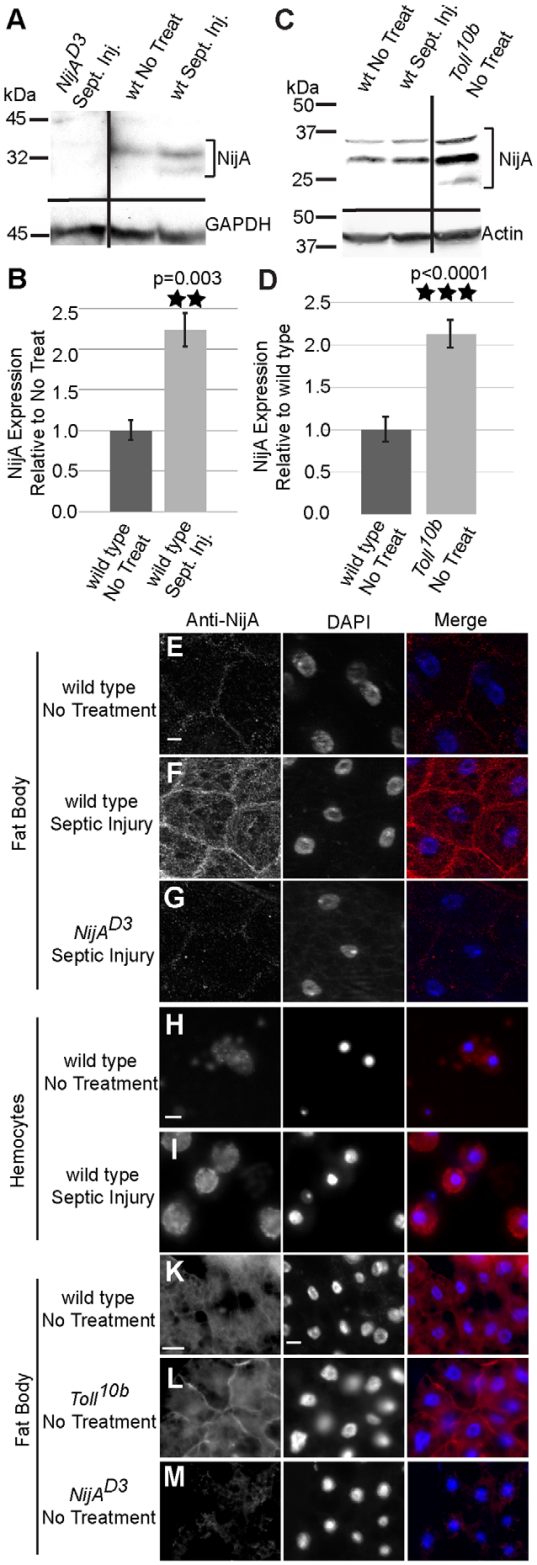
Ninjurin A protein response to septic wounding. (**A**) Western blot of whole adult male lysates probed with anti-NijA. NijA increases expression two hours after infection in adults. *NijA^D3^* null lysates demonstrate antibody specificity. Black lines indicate regions of the blot that were omitted for clarity. (**B**) Graph representing three replicates of the western blot pictured in (A). NijA levels increase significantly in adults after septic injury (p = 0.003). (**C**) Western blot of whole male larval lysates probed with anti-NijA. NijA levels do not change 2 h after septic injury in third instar larvae; in contrast larval *Toll^10b^* gain-of-function mutant larvae have increased levels of NijA protein. (**D**) Graph representing five replicates of the western blot pictured in (C). NijA levels increase significantly in constitutively activate *Toll^10b^* mutant larvae (p<0.0001). (**E–M**) Anti-NijA (red) and DAPI (blue) labeling nuclei. All scale bars are 10 µm. (**E–G**) Anti-NijA stained non-permeabilized fat bodies of male third instar larvae show an increase in NijA at the cell surface 2 h after septic injury (compare E and F). (**G**) *NijA^D3^* larvae demonstrate the NijA antibody specificity. (**H,I**) Anti-NijA stained non-permeabilized hemocytes of third instar larvae *ex-vivo* show an increase in NijA at the cell surface 2 h after septic injury. (**K–M**) Anti-NijA stained permeabilized fat bodies of male third instar larvae show increased NijA expression in gain-of-function *Toll^10b^* mutants. Error bars in (B,D) represent standard error of the mean.

Ninjurin A (NijA) is one of three *Drosophila* Ninjurin family members, and genome-wide analyses have indicated that its transcript is upregulated between 3–12 fold upon septic injury in adults or immune challenge in cultured cells [5], [10], [11]. Using a polyclonal antibody we made to the N-terminal peptide of NijA [3], we determined by western blotting that the protein levels in whole adults increase 2 h after septic injury by about two-fold, verifying the microarray studies (Fig. 1A,B). In contrast, in larvae treated with septic injuries, we did not observe an increase in NijA protein in lysates from whole animals in each of six replicates (Fig. 1C, left lanes). Because western blots of whole larvae might obscure changes in tissue-specific expression or protein localization, we compared NijA protein by immunohistochemistry in tissues from untreated larvae or larvae 2 h after septic wounding. We examined three candidate larval tissues that respond to septic wounding: fat body, hemocytes (immune cells of the blood), and epidermal wound sites. There was no change in NijA at the site of injury at the wound site (data not shown). Fat bodies are known to be heterogeneous across the tissue [16], so we reduced the variability by examining only the cells surrounding the testis; in this area NijA protein distribution was clearly altered after septic wounding in 4/4 fat bodies compared to 6 unwounded (Fig. 1E–G; p = 0.0048, Fisher's exact test). Blood cells were examined *ex vivo*, and NijA staining was altered in 9/9 samples of blood cells after septic wounding compared to 9 unwounded (Fig. 1H,I; p = 4.1×10^−5^, Fisher's exact test). In both fat and blood cells, there was a marked increase in NijA localized to the cell surface, easily observed in unpermeabilized tissue since our antibody recognizes an extracellular epitope of the NijA transmembrane protein [3]. Thus NijA protein responds to septic injury in both adults and larvae.

Since fat body and blood cells are both *Drosophila* immune organs [17], we asked whether the immune regulator Tl was capable of regulating NijA [18]. We found that whole larvae with the constitutively active *Tl^10b^* mutation have higher levels of NijA protein, even in the absence of injury (Fig. 1C, D). Anti-NijA immunostaining of the fat body indicated that NijA levels were increased in this tissue in 9/9 *Tl^10b^* mutants compared to wild type (Fig. 1K–M; p = 4.1×10^−5^, Fisher's exact test), and this Tl-mediated upregulation appears to increase NijA levels at the cell surface. The sufficiency of Tl to upregulate *NijA* in larvae is consistent with the microarray findings of De Gregorio *et al* that *spz* flies, which cannot activate the Tl pathway, also cannot upregulate *NijA*; in contrast, larvae mutant for the Imd pathway were able to upregulate *NijA* like wild type [11]. The regulation of *NijA* by the *Tl* pathway, combined with its relocalization after septic injury in the immune tissues of the blood and fat body, suggest that NijA functions in the immune system of larvae.

### *NijA* is not required for viability

**Figure 2:**
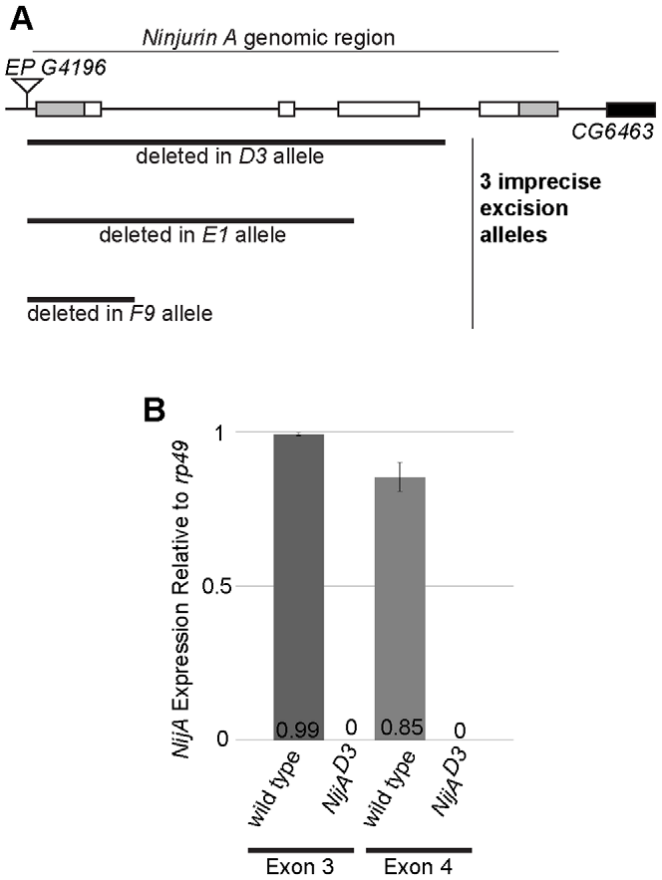
*NijA^D3^* mutants do not express mRNA from the *NijA* genomic locus. (**A**) Schematic of the *NijA* locus showing all four exons. Gray indicates untranslated regions and white indicates open reading frame. Three excision alleles (*D3, E1*, and *F9*) were generated from imprecise excisions of *EP G4196*. (**B**) qPCR data from primers specific to exon 3 (a negative control, as it is deleted in the *D3* allele) or exon 4 of *NijA*. The *NijA^D3^* mutant did not produce any detectable mRNA from exon 4 of the *NijA* locus, even though exon 4 remains in the genome, indicating that the *D3* allele is a null. Error bars represent standard error of the mean.

To understand the functional requirements for *NijA*, we made a deletion mutant by excising a *P* element at the genomic locus. Three imprecise excisions were generated that removed part of the *NijA* coding sequence: *D3, E1*, and *F9* (Fig. 2A). The *D3* allele removed the 5′ UTR and most of the coding region including the last internal methionine, suggesting that *D3* may be a null allele. To determine whether there was internal translation of the 3′ remnant of the gene in the *D3* allele, we performed quantitative PCR on the fourth exon, present in the *D3* allele, comparing its transcription level to the third exon, deleted from the *D3* allele and acting as a negative control. We found no transcription of either the third or fourth exon, confirming that the *D3* allele is a null (Fig. 2B). *NijA^D3^* homozygous mutants were viable and fertile with no obvious developmental abnormalities (data not shown). Thus *NijA* is not required for viability.

To examine the role of *NijA* in the immune system, we tested viability of *NijA^D3^* homozygous mutants after wounding or infection with gram positive or negative bacteria, but found no differences in survival or melanization (Supporting Information S1 and data not shown). The ability of *NijA^D3^* mutants to mount an antimicrobial peptide response after septic injury was examined by measuring Drosomycin (Drs) or Drosocin (Dro), both targets of the Tl pathway [18]. Both antimicrobial peptides were elevated in the *NijA^D3^* mutant in a manner not significantly different from wild type (Supporting Information S2 A,B). To further assess a potential role for *NijA* in Tl signaling, we performed an epistasis test to ask whether *NijA* is required for the upregulation of a Tl pathway target when *Tl* is genetically activated by mutation in the fat body (by driving *Tl^10b^* with the *c564*-*GAL4* driver). Examining the levels of Drs, we found that Drs elevation was similar after septic wounding of wild type or genetic activation of *Tl^10b^* in the fat body, and these levels were not affected by the *NijA^D3^* mutation in *Tl^10b^ NijA* double mutants (Supporting Information S2C). To examine the cellular immune response we assayed phagocytosis and found that the capacity of hemocytes or S2 cells to phagocytose labeled *E. coli* did not depend on *NijA*, although hemocyte phagocytosis was sensitive to a dominant locus on the *NijA^D3^* chromosome (Supporting Information S3). Similarly, a dominant locus on the *NijA^D3^* chromosome obscured our ability to assess a homozygous mutant phenotype in response to starvation (Supporting Information S4). Thus we were unable to identify a function for *NijA* using loss-of-function approaches.

### Ectopic expression of *NijA* induces nonapoptotic cell death

Because NijA is upregulated on septic injury, we focused on analyzing its function by ectopically upregulating *NijA* using the *GAL4/UAS* system for a gain-of-function approach. Under the ubiquitously expressed *tubulin* and *actin* drivers, we found that embryos died with morphological abnormalities around the time of cellularization (data not shown), which is also when zygotic transcription begins [19], [20]. Lethality was also observed when we expressed *NijA* with *A58-GAL4* in the larval epidermis [21] or in most blood cells with *He-Gal4* [22] (data not shown). Attempts to control the onset of lethality by using the conditional *GAL80^ts^* inhibitor [23] or the inducible *GeneSwitch-GAL4* driver [24] both failed because even under restrictive conditions (18° or absence of RU486, respectively) leaky expression of *NijA* still caused lethality (data not shown). We expressed *NijA* in a tissue not required for viability, the eye, with *GMR-GAL4* and *ey-GAL4*; to our surprise, *NijA* expression with each driver was pupal lethal (Table 1 and data not shown). Expression of *NijA* with *hml-GAL4*, expressed in differentiated blood cells of the larval lymph gland and in circulation [25], [26], was not lethal and allowed us to ask about the cellular consequences of NijA overexpression.

**Table 1:** Organismal death resulting from GMR-driven expression of a cell-death gene is not dependent on the gene acting non-autonomously.

	# *GMR>UAS*	Expected	
cross	progeny observed	Mendelian #	X^2^ value
		if viable	
*GMR-GAL4/CyO*			
*x*	3/338 (0.89%)	85.5/338 (25%)	104.8 (p<0.001)
*UAS-NijA/TM3*			
*GMR-GAL4/CyO*			
*x*	3/175 (1.7%)	58.3/175 (33%)	78.7 (p<0.001)
*UAS-hid/CyO*			

**Figure 3:**
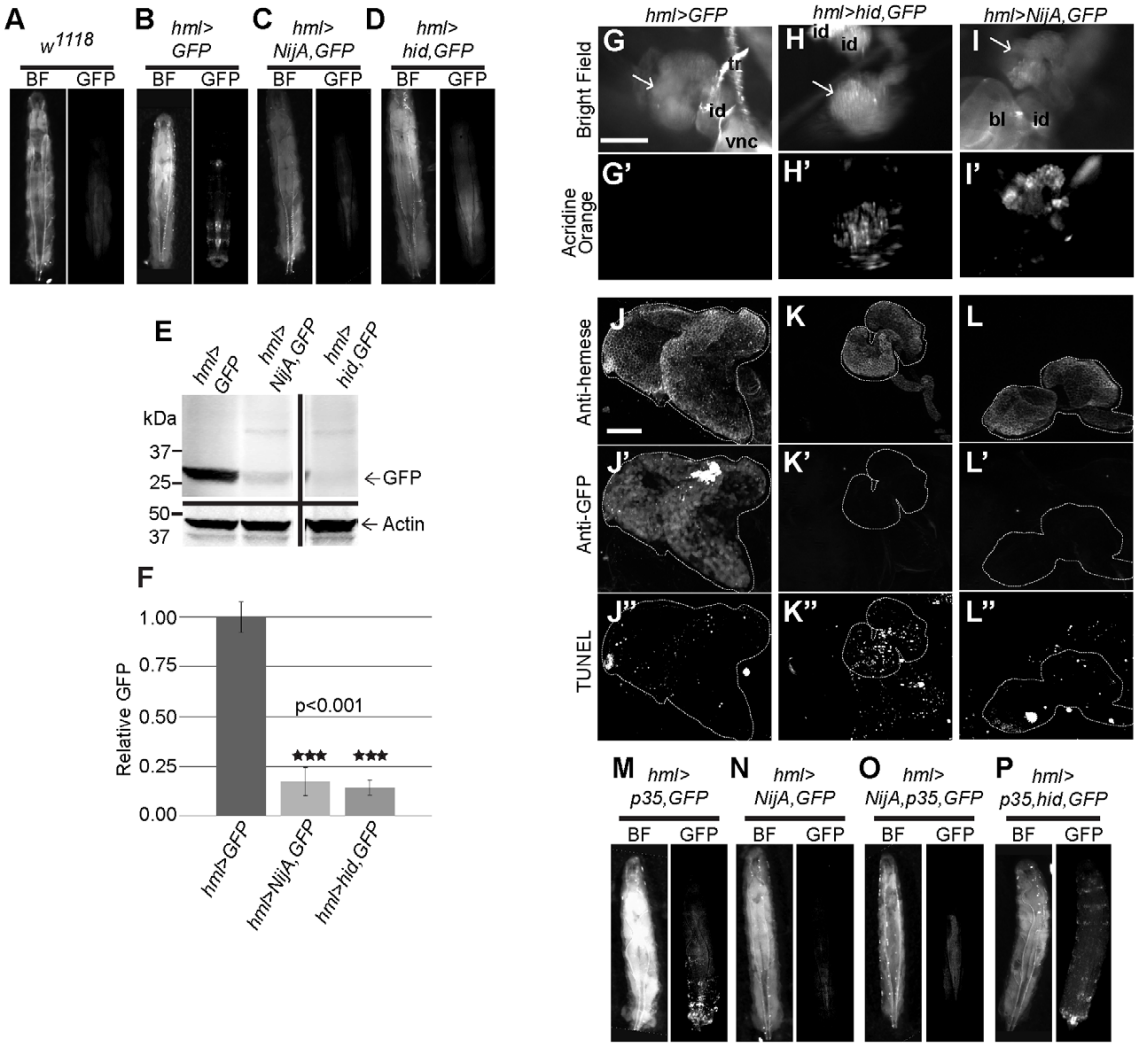
Ninjurin A over-expression in the lymph gland causes nonapoptotic cell death. (**A**) Wild-type *w^1118^* larva demonstrating background autofluorescence. (**B**) *hml>GFP* larva with GFP-positive differentiated hemocytes along posterior body wall and in lymph gland. (**C**) *hml>NijA,GFP* larva lacked GFP-positive cells. (**D**) *hml>hid,GFP* larva, a cell-death positive control, lacked GFP-positive cells. (**E**) Western blot of whole larval lysates probed with anti-GFP. *hml>NijA,GFP* and *hml>hid,GFP* larvae were devoid of GFP. (**F**) Quantification of three western blots probed for anti-GFP as in (E). GFP is virtually absent from *hml>NijA,GFP* and *hml>hid,GFP* larvae. Error bars represent standard error of the mean. (**G–I′**) Live partially dissected 3^rd^ instar larval lymph glands (arrows) were stained with acridine orange to detect cell death. Scale bars are 200 µM. tr: trachea; id: imaginal disc; vnc: ventral nerve cord; bl: brain lobe. (**G′**) *hml>GFP* larval glands did not stain with acridine orange. (**H′**) *hml>hid,GFP* glands, a cell-death positive control, stained with acridine orange. (**I′**) *hml>Nij*A,GFP glands stained with acridine orange, demonstrating that *NijA* induced cell death. (**J–L″**) Larval lymph glands were fixed, TUNEL labeled, and antibody stained. Scale bars are 50 µm. (**J–L**) Anti-Hemese staining labeled the lymph glands. (**J′–L′**) Anti-GFP staining shows no GFP-positive (hml+) hemocytes in *hml>hid,GFP* (K′) or *hml>NijA,GFP* (L′) larval glands. (**J″–L″**) TUNEL-labeled glands. (**J″**) Few TUNEL positive cells in *hml>GFP* negative control glands. (**K″**) Many TUNEL positive cells in *hml>hid,GFP* positive control glands. (**L″**) Few TUNEL positive-cells in the *hml>NijA,GFP* glands, indicating that *NijA* does not induce apoptosis. (**M**) Larvae expressing the apoptotic inhibitor p35 (*hml>p35,GFP)* displayed GFP-positive hemocytes similar to *hml>GFP* in (B). (**N–O**) p35 did not inhibit the NijA-induced loss of the GFP-positive cells in *hml>NijA,p35,GFP* larvae. (**P**) p35 inhibited the hid-induced loss of the GFP-positive cells in *hml>hid,p35,GFP* larvae, a positive control for p35 inhibition. In (A–D, M–P), anterior is up.

*Hml* expressing cells, visualized by *Hml>GFP*, can be observed under the cuticle of whole third instar larvae (Fig. 3A,B); however, when *NijA* is co-expressed, the cells appear to be absent (Fig. 3C). To compare the effects of *NijA* to a known apoptosis inducer, we drove the expression of *hid* with *hml-GAL4*, and found a similar loss of GFP-labeled cells (Fig. 3D). The similarity of these results suggested that *NijA* induces cell death in differentiated blood cells. We confirmed by quantitative Western blotting that GFP was absent from animals co-expressing *GFP* and either *NijA* or *hid* (Fig. 3E,F). This lack of GFP indicated that larvae were missing nearly all *Hml*-expressing cells, both in circulation and in the lymph gland, the site of hematopoiesis in larvae.

We examined dissected lymph glands expressing *NijA*. Although 7/7 control (*hml>GFP*) lymph glands did not stain with acridine orange, which can only permeate dead cells, in contrast all lymph glands expressing either *NijA* or the apoptosis inducer *hid* stained brightly with acridine orange (7 of each genotype, compare Fig. 3G–I′). The dying cells appeared to be restricted to differentiated cells that express *hml*, as undifferentiated hemocytes expressing *hemese* but not *hml* were still present in lymph glands expressing *hml>NijA* or *hml>hid* (Fig. 2J–L). We were able to detect little to no GFP signal with an anti-GFP antibody in the lymph glands of *hml>NijA,GFP* or *hml>hid,GFP* animals (Fig. 3J′–L′), consistent with the dramatic decrease in GFP expression in whole animals by western blot analysis (Fig. 3E–F). Interestingly, *NijA* was not required for developmentally regulated apoptosis, however, as apoptosis in the stage 10 embryonic head region occurs normally in a *NijA^D3^* mutant with no zygotic or maternal *NijA* (Supporting Information S5). Thus *NijA* induces cell death when ectopically expressed.

To examine whether NijA induces cell death via apoptosis, we examined lymph glands by TUNEL staining, a nuclear label for apoptotic cells. We compared lymph glands expressing *GFP* and *NijA* to glands expressing only the *GFP* marker as a negative control, and to glands expressing *GFP* and the apoptosis-inducer *hid* as a positive control. We counted the number of TUNEL positive nuclei in primary lymph gland lobes: fewer than 30 nuclei was considered background, and more than 30 (often uncountable) was considered apoptotic. By these criteria, we found that 10/11 *hid*-expressing lymph glands were apoptotic, whereas 22/23 GFP lymph glands were TUNEL-negative and 9/13 *NijA*-expressing lymph glands were TUNEL-negative (Fig. 3J″–L″). The penetrance of apoptosis in *NijA* lymph glands is significantly different from *hid* lymph glands by Fisher's exact test (p = 0.004). Some of the unexpected variability in our *NijA* overexpressing lymph glands may be an artifact of our cut-offs, as 3 of the 4 *NijA* expressing lymph glands scored as “apoptotic” qualitatively appeared to have fewer TUNEL positive cells than the *hid* expressing glands; another possibility is that there is significant cross-talk between cell death pathways (see Discussion).

As a second independent assay to assess whether *NijA* induces apoptotic or nonapoptotic cell death, we co-expressed *NijA* with *p35*, an apoptotic inhibitor that blocks cell death when co-expressed with *reaper* or *hid* [27], [28]. Examining GFP-labeled hemocytes in live animals, we found that p35 inhibited Hid-induced cell death as expected, but importantly p35 did not inhibit NijA-induced cell death (Fig. 3M–P). Our data indicate that *NijA* induces a nonapoptotic form of cell death, as dying cells do not label with TUNEL and cell death is not inhibited by p35.

### NijA appears to kill cells in a cell-autonomous manner

Because the expression of NijA in the eye with *ey-GAL4* or *GMR-GAL4* was lethal to the animal, we asked whether this phenotype represented tissue nonautonomous cell death, *i.e.*, if NijA expressed in the eye disc was effectively instructing tissues outside the eye to die. Alternatively, it was possible that even the autonomous destruction of a large tissue may release toxic factors that could cause animal lethality. To determine if we could assess tissue autonomy in this assay, we expressed *hid*, which is known to be an autonomous cell-death gene [27] also under *GMR-GAL4* and found that the overexpression of *hid* caused organismal lethality with very few escapers (Table 1 and data not shown). Either *GMR-GAL4* is not eye-specific or the massive developmentally-inappropriate induction of cell death is sufficient to cause organismal death; in either case this assay cannot indicate the autonomy of NijA-induced cell death.

**Figure 4:**
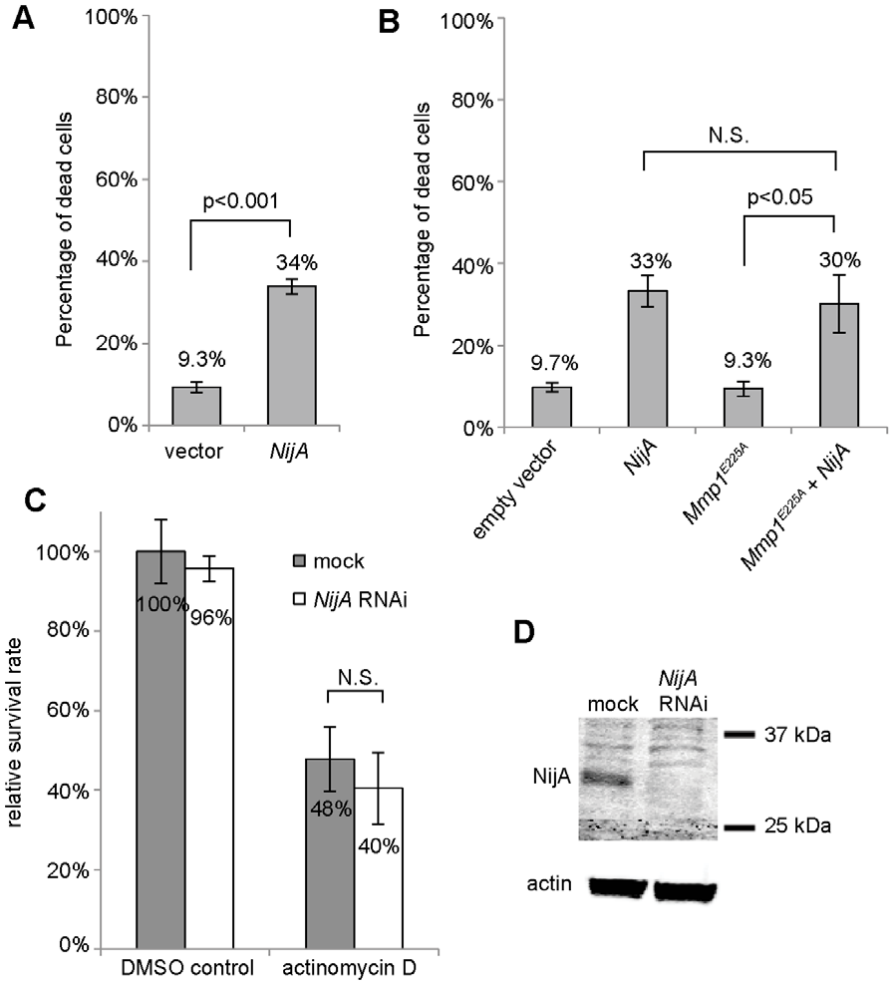
NijA induces cell death in *Drosophila* S2 cell culture. (**A**) *NijA* expression kills S2 cells. Cells were transiently transfected with *pRmHa3* empty vector or *pRmHa3-NijA* and induced with copper for 48 h. The percentage of dead cells was determined by dividing the number of trypan blue positive cells by that of total cells counted for each sample. Data from 8 experiments are shown. Error bars indicate S.E.M, and Student's T test was used to calculate p value. (**B**) Mmp1 activity is not required for *NijA*-induced cell death. Cells were transiently transfected and induced for 48 h. *Mmp1^E255A^* is a dominant-negative catalytically inactive mutant of *Mmp1*. Data from 4 experiments are shown. (**C**) *NijA* is not required for actinomycin D-induced apoptosis. Cells were treated with *NijA* dsRNA or no dsRNA (mock) for 48 h, then incubated with 100 nM actinomycin D for 6 h. Trypan blue staining was used to determine cell survival, which was normalized to the untreated (DMSO), wild-type (mock) sample. Data from 4 experiments are shown. (**D**) Western blot showing the NijA protein levels in mock and *NijA* dsRNA-treated cells. Actin was used as the loading control.

As another means to assess the autonomy of NijA-induced cell death, we turned to cell culture. We previously reported that when overexpressed in cultured *Drosophila* S2 cells, NijA inhibits cell adhesion in a nonautonomous manner within a few hours of its induction. This NijA-mediated phenotype was dependent on the activity of the protease Mmp1, and the NijA ectodomain was sufficient to release adhesion even when Mmp1 proteolysis was inhibited [3]. We revisited these experiments and found that when S2 cells express *NijA* for longer periods, the cells died as measured by trypan blue exclusion: after 24 h, 20.2% of cell had died (not shown), and after 48 hours 33% of cells had died, a significant increase over the background death rate of about 9% in non-expressing cells (Fig. 4A). Interestingly, like in whole flies, *NijA* was not required for apoptosis, as the apoptosis-inducer actinomycin D [29], [30] was able to induce death at similar levels in wild-type and *NijA* knock-down cells (Fig. 4C,D), indicating that *NijA* is not an essential component of the cell death machinery. In contrast to our previous adhesion results, NijA-induced death does not require Mmp1 (Fig. 4B). Similarly, we found that the NijA ectodomain is not sufficient to trigger cell death, even though we were able to detect the tagged ectodomain in cells, cell lysates, and in the culture medium (Supporting Information S6). These Mmp1 and NijA ectodomain results suggested that NijA may induce death in a cell-autonomous manner.

**Figure 5:**
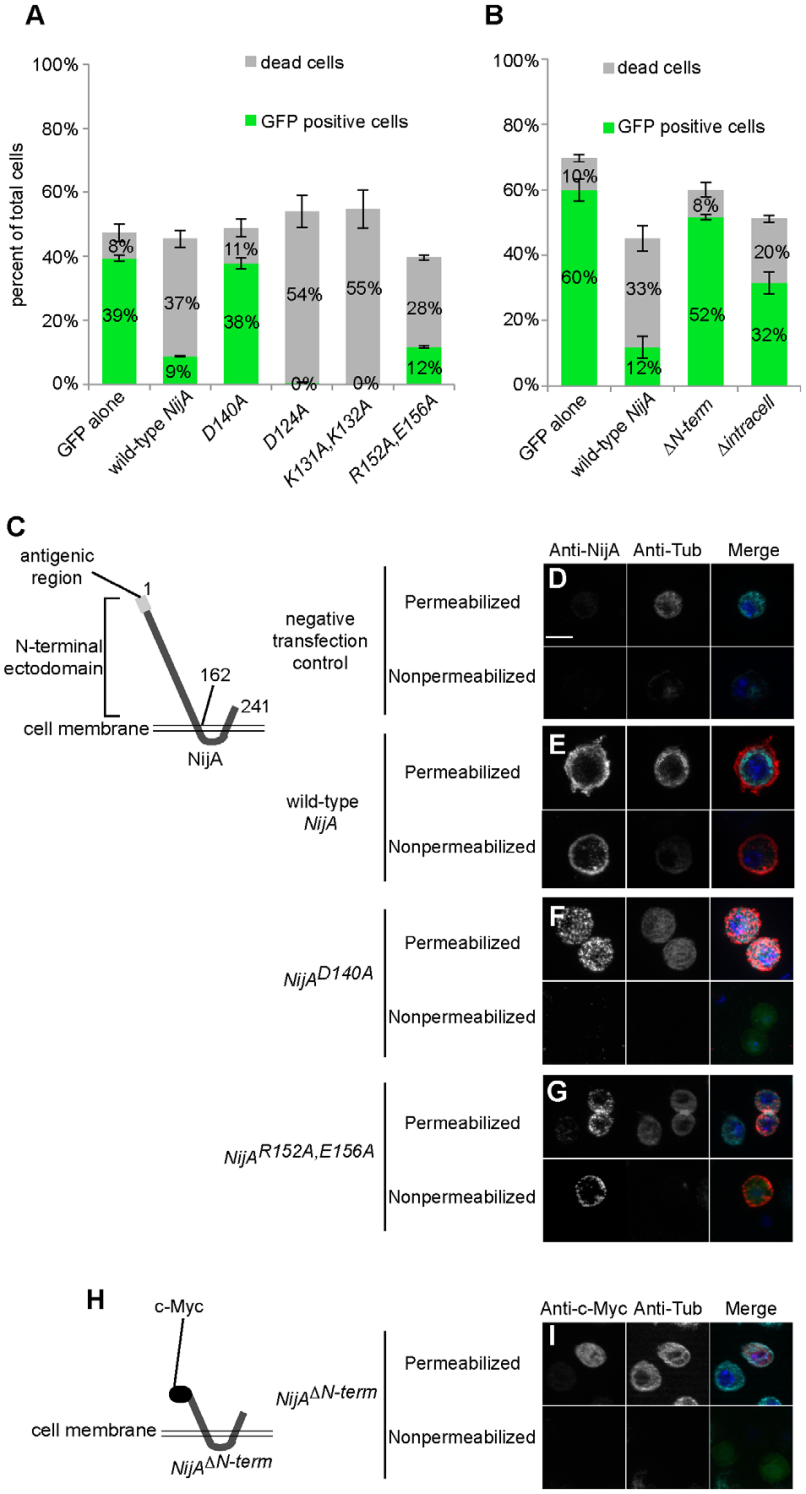
NijA appears to kill in a cell-autonomous manner. (**A–B**) Cells were transiently co-transfected with *pRmHa3-GFP* and various mutants of *pRmHa3-NijA* as indicated under each column; mock is empty *pRmHa3* vector. 48 h after induction, viability was assessed by trypan blue staining, and transfection status was assessed as GFP fluorescence. Wild-type *NijA* and most *NijA* mutants killed cells, whereas the mock control, the *D140A* mutant, and the N-terminal deletion (B) showed low levels of cell death. The sum of transfected live cells (GFP+) plus dead cells was relatively constant across samples despite the augmented or compromised capacity to kill cells, indicating that NijA kills the cell it transfects but not others. Data from 3 replicates are shown. Error bars indicate S.E.M. (**C**) Schematic showing topology of NijA (form A) protein and the extracellular region recognized by our polyclonal antibody. Amino acid residue numbers are indicated. (**D–G,I**) Immunofluorescence localization of wild-type NijA or NijA mutant forms expressed in S2 cells and stained with anti-NijA (D–G) or anti-c-Myc (I), both extracellular epitopes. For each construct, staining was performed on permeabilized cells to show NijA protein levels, and on unpermeabilized cells to show NijA cell-surface localization. Permeabilization status was verified by anti-tubulin staining. The merge image combines images for NijA (red), tubulin (cyan), DAPI (blue) and GFP fluorescence as a transfection control (green). Bar: 10 µm. (**H**) Diagram showing placement of the myc epitope for (I), necessary because the NijA antigenic region was deleted in this mutant.

To examine the autonomy of Ninjurin-induced cell death, we considered testing conditioned media, but we were concerned that dying cells may release toxic factors into the media even if the death were cell autonomous, similar to our results in whole animals. Instead, we chose to examine the relationship between NijA transfection status labeled by GFP and cell death measured by trypan blue exclusion. We co-transfected NijA and GFP expression vectors and counted the number of GFP-expressing cells remaining after 48 h induction, as transfected cells are expected to take up both vectors simultaneously (Fig. 5). When transfected with only the *GFP* plasmid, 39% of cells expressed GFP, and 8% of cells were dead as measured by trypan blue exclusion (Fig. 5A), consistent with the baseline rate of cell death we measured in these cultures (Fig. 4). In contrast, when cells were co-transfected with *NijA* and *GFP*, only 9% of cells expressed GFP, and 37% of the cultured cells were dead as measured by trypan blue exclusion (Fig. 5A). Thus it appeared that *NijA*-expressing cells were much more likely to die than their non-transfected neighbors. We continued this analysis to ask which residues were important for inducing cell death, examining four site-directed *NijA* mutants generated by alanine-scanning [31], in which charged residues in the ectodomain were replaced with alanines (Fig. 5A,C). We found that D140 was absolutely required, as this point mutation ablated NijA's ability to induce death; importantly the D140A mutant protein was not able to localize correctly to the cell surface (Fig. 5D–F), suggesting that cell-surface localization is critical for inducing death. Surprisingly, two mutants seemed to increase the potency of NijA, as D124A or the double mutant K131A, K132A were more toxic to cells than wild-type NijA, resulting in no GFP-positive cells; yet because the fraction of dead cells was similar to the sum of the baseline death rate plus the transfection rate, it appears that this overactive toxic mutant still killed in a cell-autonomous manner. The double-mutant R152A, E156A killed cells similarly to wild-type NijA (Fig. 5A), and the mutant protein localized at the cell surface similarly to wild-type NijA (Fig. 5G). In a separate experiment, we asked which domains were required to induce death (Fig. 5B). The N-terminal ectodomain was required, as a deletion removing it (*NijA^ΔN-term^*) induced only 8% death with 52% of the cells expressing GFP, similar to the GFP-alone controls. The ectodomain was also required for localization of NijA to the cell surface, as the NijA^ΔN-term^ protein (carrying a myc epitope at the new N-terminus) was not detectable in unpermeabilized cells (Fig. 5I). The predicted 20-amino acid intracellular domain was partially required, as a mutant replacing most of the intracellular sequence (*NijA^Δintracell^*) with the myc epitope gave an intermediate level of death, with 20% dead cells and 32% GFP-expressing cells. Taken together, these data strongly suggest that NijA induces death in a cell-autonomous manner, requiring cell surface localization to kill cells.

**Figure 6:**
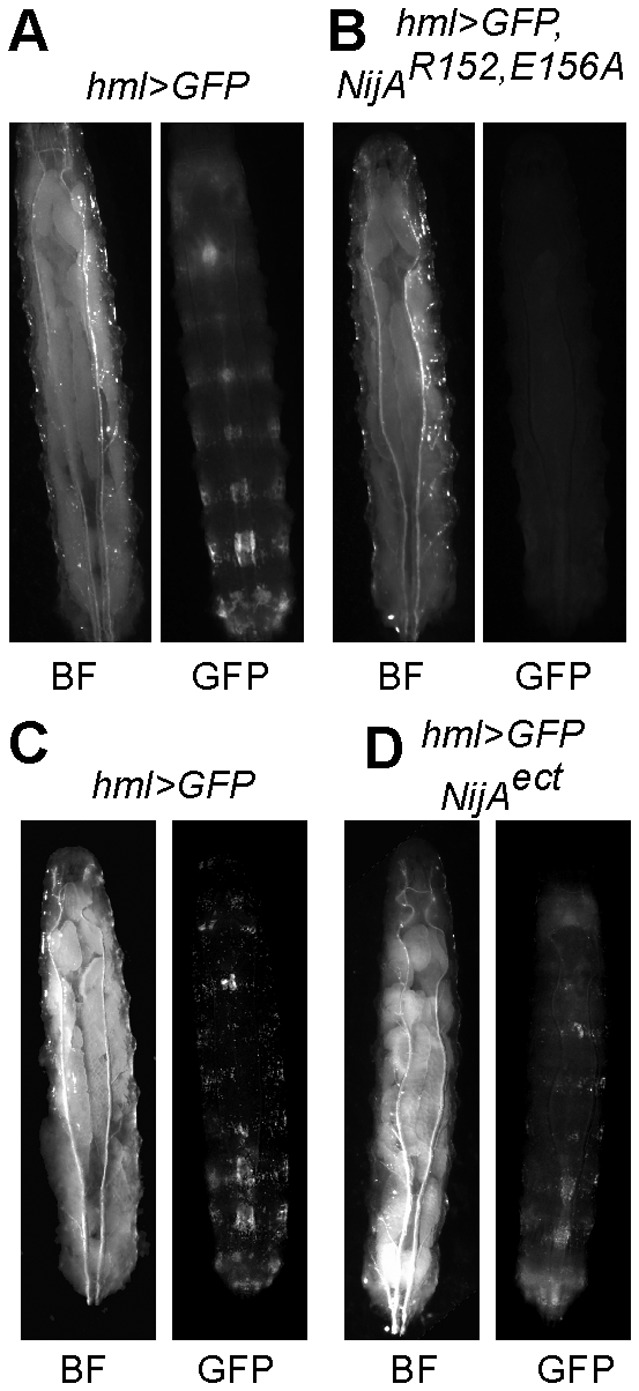
*NijA* mutants behave *in vivo* as they do in cell culture. (**A**) Third instar *hml>GFP,NijA^152156A^* larvae were devoid of GFP-positive hemocytes, indicating that these mutants were capable of inducing cell death and that polar amino acids 152 and 156 were not required for cell death. (**B**) *hml>NijA^ect^,GFP* larvae displayed visible GFP-positive hemocytes on the body wall, suggesting that NijA^ect^ was not sufficient to induce cell death.

To determine if these mechanisms applied to NijA cell death induction *in vivo*, we transformed flies with GAL4-inducible constructs encoding the *NijA* double mutant *R152A, E156A* and the NijA ectodomain (*UAS-NijA^ect^*). As in cell culture, the double mutant phenocopied wild-type *NijA*, whereas the ectodomain alone did not induce cell death comparable to wild-type *NijA* (Fig. 6). We conclude that when expressed at high levels, *NijA* kills cells by a nonapoptotic mechanism, likely in a cell autonomous manner.

## Discussion

In the present study, we found that *Drosophila* NijA is upregulated or relocalized in tissues of the immune system upon septic injury. The NijA upregulation observed in adults may result in more protein localized to the cell surface, a similar effect to the relocalization to the cell surface observed in larvae after septic injury. NijA protein levels are upregulated via the Tl pathway, an established immunoregulatory pathway, and in activated Tl mutants, there is more NijA observed at the surface of fat body cells, supporting the idea that NijA upregulation and relocalization are functionally similar immune responses. In whole-animal genetic experiments we found that the ectopic upregulation of NijA induces cell death characterized by acridine orange staining and tissue loss. However, NijA-induced cell death is not associated with DNA fragmentation, as assessed by TUNEL labeling, and is not suppressible by p35, an apoptotic inhibitor. These data indicate that NijA induces nonapoptotic cell death. Similar death phenotypes were observed both in tissues of whole larvae and in cultured S2 cells, and the cell death appears to be autonomous. In cultured cells, *NijA* mutants that cannot localize to the cell surface also cannot induce cell death, suggesting that *in vivo* the observed increase of NijA levels at the cell surface after septic injury may be critical to NijA function.

Originally identified as gene products upregulated on nerve injury, Ninjurins have been characterized as adhesion molecules [1], [13], [14], [15], anti-adhesion signals [3], and mediators of cell cycle regulation [32]. This lack of consensus likely stems from the fact that these studies have been performed in cultured cells. There is scant information about Ninjurin function *in viv*o, in part because animal studies have focused almost exclusively on expression analysis [1], [2], [3], [14], [15]. The only study investigating Ninjurin function *in vivo* was performed with neutralizing antibodies to Ninjurin1 during rat ocular development [14], and it found that Ninjurin antibodies slowed the regression of the hyaloid vasculature, an embryonic tissue that is removed by cell death during development.

Our *in vivo* gain-of-function data suggests that Ninjurins promote nonapoptotic cell death. In addition to apoptosis, the other main types of programmed cell death are autophagy and programmed necrosis (sometimes called necroptosis) [33]. Autophagy, a process in which the cell digests its own components, can regulate a variety of cellular processes including viral clearance and signal transduction [34], [35]. Autophagy can result in cell survival in response to stress or can lead to cell death [36]. The death outcome utilizes components of the apoptotic machinery [37]. The differences between autophagic death and apoptosis are still being elucidated, and it is likely that in some contexts they can compensate for each other if one death mechanism fails [38], [39]. The pathways leading to programmed necrosis also have considerable crosstalk with the pathways leading to apoptosis, and they are believed to inhibit each other [40]. Unfortunately, programmed necrosis has not been characterized in *Drosophila*, although it may be induced genetically [41], [42]. We speculate that the interplay between these different types of cell death may explain why we observe no cell-death phenotypes in the *NijA* null mutants. Because cell death mechanism are known to compensate for each other, it is possible that in our *NijA* null mutants, cells that would have been killed by a NijA-dependent mechanism are now killed by another cell-death mechanism. Of note, genome-wide expression studies report that NijA is expressed during metamorphosis at levels ∼12-fold higher than during any other time in the *Drosophila* life cycle ([43] as reported at flybase.org), suggesting that NijA may function during cell and tissue death in development. Interestingly, several Toll family members (Toll, 18-wheeler/tollo, Toll-6, and Toll-7) are also highly expressed during metamorphosis as well [44], and our data demonstrates that Tl can upregulate NijA.

Another possibility to explain the lack of cell death or other phenotypes in our *NijA* null mutants is genetic redundancy among the three Drosophila Ninjurin genes, *NijA, NijB*, and *NijC*. However, expression data does not support the idea of Ninjurin family redundancy. Genome-wide expression data sets, examining developmental timing and tissue-specific expression of all genes, indicate that *NijA, NijB*, and *NijC* are not expressed in similar times or tissues in developing flies ([43], [45], [46] as reported at flybase.org). Nevertheless, it is possible that analysis of double and triple mutants may uncover redundant Ninjurin functions. In our preliminary examination of *NijA, NijC* double mutants we have not found any obvious developmental abnormalities (X. Wang and A. Page-McCaw, unpubl.).

Our data suggest that Ninjurin may participate in an immune response that promotes cell death. Although cell death is critical in the mammalian immune system [40], in Drosophila it is unclear why immune tissues would initiate death in response to immune challenge. One possibility is that NijA is required not for the initial immune response, but rather for resolving the immune response once a pathogen has been neutralized. In mammals, superfluous neutrophils are cleared by cell death after resolution of an immune challenge [47], and if neutrophils are not cleared excessive inflammation can result in damage to healthy tissue [48]. In Drosophila, there is a dramatic increase in circulating hemocytes after immune challenge [49], and it is not clear if these hemocytes persist after the challenge has been eliminated; it is possible that NiA may participate in their clearance. Another possibility is that increased NijA primes cells to die in an orderly manner if they should become injured in the course of an infection. A third possibility is based on the idea that physiological increases of NijA in response to septic injury are significantly smaller than those in our genetic overexpression system; perhaps on immune challenge, moderate levels of NijA initiate an autophagic mechanism of pathogen clearance from cells, but at physiologic levels NijA does not promote cell death at all.

Our genetic gain-of-function studies indicate that NijA induces cell death in a cell-autonomous manner. This is different from the nonautonomous apoptotic cell death observed in endothelial cells adjacent to Ninjurin1-positive macrophages that was interpreted as a result of altered adhesion [14], and it is different from the cell nonautonomous loss-of-adhesion signaling we observed in cultured cells [3]. It is possible that in different contexts Ninjurins can act either autonomously or nonautonomously, as their cell-membrane location would allow them to relay information from outside to inside the cell, or allow them to signal via their extracellular domain to other cells. Our study highlights a novel and potentially medially important role for the conserved Ninjurin family in inducing cell death.

## Materials and Methods

### *Drosophila* genetics and imaging

Unless otherwise noted flies were raised at 25° under standard conditions. *Hml-GAL4, UAS-GFP* and *UAS-hid* were from J. Royet [25]; *C564-GAL4* and *Tl^10b^* were from K.V. Anderson [50], [51]; *UAS-Tl^10b^* was from S. Cherry [52]. The *EP* element *G4196* was generated by Genexel (Korea) and deposited at the Bloomington *Drosophila* Stock Center. The site of the insertion was determined by sequencing genomic DNA to be 51 bp upstream of the transcription start site. The insertion line was outcrossed 3 times to *w^1118^* before excising the transposon. 60 excision lines were screened in pools of five by PCR amplification of a 3561 bp genomic fragment surrounding the P insertion site; from these three imprecise excisions (shown in Figure 2) were identified by gel electrophoresis of PCR products. The *UAS-NijA* line was generated by ligating the cDNA *RE5744* (Berkeley *Drosophila* Genome Project) corresponding to NijA-RA, into the *pUAST* vector at the EcoR1 and BamH1 sites. The fly transformation vectors *UAS-NijA^ect^* and *UAS-NijA^R152A,E156A^* were generated by ligating the inserts from the corresponding *RmHa3* plasmids (see below) into *pUAST*. Transformants were generated by Genetic Services Inc (Cambridge, MA). We examined two independent transformants of the *UAS-NijA* and *UAS-NijA^ect^*, and in both cases we saw comparable results.

For *hml>GFP* analysis in whole larvae, third instars were selected from the food of a healthy vial, washed in sterile water to remove debris, and placed on a grape juice plate. GFP-labeled blood cells were scored in live larvae under a Zeiss LumarV12 fluorescence stereomicroscope prior to heat-killing for imaging. A minimum of 20 animals were scored per genotype. For imaging, each larva was placed in 20 µl of PBS on a cover slip, and heated for 5 sec at 95°C to kill the larvae. Larvae were immediately imaged by bright field and epifluorescence with a Zeiss 0.8× Neolumar objective, on magnification setting 64×. Images were cropped and edited using Adobe Photoshop.

For imaging lymph glands live-stained with acridine orange, third instar larvae were selected from the food of a healthy vial and washed in sterile water to remove debris. Each larva was placed in a 50 µl drop of freshly diluted 1.6×10^−6^ M acridine orange in *Drosophila* Ringer's solution on a Sylgard dissection plate. The dorsal cuticle was carefully torn away from posterior to the anterior to expose the internal organs. The flap of dorsal cuticle was pinned to the plate, and the fat surrounding the dorsal vessel quickly cleared away for imaging. Just prior to imaging the acridine orange solution was removed and replaced with *Drosophila* Ringer's solution. Bright field and epifluorescence images were taken on a Zeiss LumarV12 with a 1.5× Neolumar objective and processed using Adobe Photoshop.

### Western blotting and antibody staining

For Western blots, lysates were made by mechanically grinding samples on ice in Laemmli buffer and heating at 95°C for 5 min. Blots were probed with guinea pig anti-NijA at 1∶1500 [3], mouse anti-GAPDH at 1∶2000 (IMGENEX, #IMG3073), mouse anti-Actin at 1∶2000 (Abcam, #ab6276), and rabbit anti-GFP at 1∶2000 (Abcam, #ab6556). Detection was performed by either HRP-mediated chemiluminescence or fluorescence imaging (Licor, Odyessy). Figure 1A and Supplementary Figure 3C were probed with HRP-labeled goat anti-guinea pig at 1∶5000 (Santa Cruz) and HRP-labeled goat anti-mouse at 1∶5000 (Jackson Immuno-research) secondary antibodies. Figures 1C, 2E, and 4D were probed with IRDye 680-labeled donkey anti-mouse, IRDye 800CW-labeled donkey anti-guinea pig, IRDye 680-labeled donkey anti-rabbit, or IRDye 800CW-labeled donkey anti-mouse, all diluted 1∶5000 (Licor). Data was quantified using the ImageJ software. Blots were cropped and edited using Adobe Photoshop.

For antibody staining, samples were dissected, fixed in 4% paraformaldehyde in phosphate buffered saline (PBS) for 20 mins, and then blocked for 30 mins in 1% bovine albumin serum (BSA) in PBS+0.2%Tween (PBST) for permeabilized samples or in 5% normal goat serum (NGS) in PBS for non-permeabilized samples. Primary antibodies were diluted in blocking reagent and incubated with sample overnight at 4°C. Primary antibodies used were guinea pig anti-NijA at 1∶100 [3], mouse IgG2a anti-hemese at 1∶100 (from Istvan Ando [53]), and rabbit anti-GFP at 1∶50 (Abcam, #ab6556). Samples were washed and labeled with secondary antibody for 2 h in the dark at room temperature. Secondary antibodies used were Cy3-labeled goat anti-guinea pig, DyLight 649-labeled goat anti-mouse, and FITC-labeled goat anti-rabbit, all diluted 1∶500 (Jackson ImmunoResearch). TUNEL labeling was performed according to the manufacturer's directions (Roche *In situ* cell death detection Kit TMR red). Tissues were mounted in Vectashield (Vector Lab) and imaged on a Zeiss Imager M2 with Apotome. Images are 2D projections of Z-sections. Projections were generated by the ImageJ software, and images were cropped and edited using Adobe Photoshop.

Hemocytes were recovered for *ex vivo* staining as described [54]. In brief, the posterior end of a clean larva was bled onto a glass slide, and the hemolymph was recovered with a pulled glass needle. (To control the needle suction with a P20 pipettor, the dull end of the pulled needle was attached to the small end of a P20 tip by melting the plastic tip and sealing with nail polish.) The hemolymph was transferred to a Multitest slide (MP Biomedicals) in 10 ul of PBS, and the hemocytes were allowed to settle for 10 mins. Hemocytes were fixed for 7 mins in 4% paraformaldehyde in PBS, rinsed briefly in PBS, and then blocked for 15 mins in 1% BSA in PBST. The primary antibody was applied to the samples for 2 h while the slide was in a humidified chamber. The samples were washed in several changes of PBST, and the secondary antibody was applied for 1 h in a dark humidified chamber. The samples were washed several times in PBST, and once in PBS prior to mounting in Vectasheild mounting media for viewing on a Zeiss Imager M2 with a Plan Neofluor 40× oil objective.

### qPCR Analysis

Total RNA was extracted from whole third instar males using the TRIzol reagent (Ambion) according to the manufacture's directions. Only males were used because of sex-specific differences in antimicrobial peptides [55]. Total RNA extracts were treated with DNase to remove contaminating DNA with the TURBO DNA-free kit (Ambion). 800 ng of total DNase-treated RNA was reverse-transcribed into cDNA pools using the iScript cDNA Synthesis Kit (BioRad) according to the manufacture's directions in an Eppendorf AG 22331 Hamburg Thermocycler. 2 µl of the cDNA pools were primed with validated primers sets for NijA^Exon 3^ (Fwd:AACTGTTGGAGGCAACGGAG, Rev:AAAGGAGAAACTGGGTCGTCTT. R^2^<0.99), NijA^Exon 4^ (Fwd:GCGTGGGCCTTATATTGATG, Rev:TGTTCGCCCGGCAGATAT, R^2^<0.99), and rp49 (R^2^<0.99) [56]. qPCR reactions were run using the SSO Advanced SYBR Green Super Mix (BioRAD) for SYBR green chemistry according to the manufacture's directions in a CFX96 Real-Time C1000 Thermocycler (BioRad). Resulting Ct values were analyzed in Microsoft Excel. Ct values were fit to a standard dilution curve for correction to primer efficiency and then normalized to the *rp49* housekeeping gene. Three replicates were performed for each condition.

### Cell culture and transient transfection

*Drosophila* S2 cells were obtained from the Drosophila Genomics Resources Center (Bloomington, IN) and maintained at 27°C in Schneider's *Drosophila* medium (Gibco) containing 10% heat inactivated Fetal Bovine Serum (Gibco, 16140) and 100 U/ml penicillin/streptomycin. For transient transfection, 3×10 ^5^cells were seeded per well in 750 µl of complete medium in a 24-well plate. The next day, cells were transfected with 2 µg plasmid DNA/well (1 µg DNA/well for co-transfected GFP plasmids) using the calcium phosphate method according to manufacturer's protocols (Invitrogen). *NijA-RA* wild-type, mutant, deleted, tagged, and ectodomain constructs were each cloned into *pRmHa3* for inducible expression from the metallothionine promoter; the wild-type and ectodomain constructs were described in [3]. The *Mmp1^E225A^* mutant, also in *pRmHa3*, was in splice isoform 1 (*Mmp1-RD*) and encodes an inactivating mutation at the catalytic core rendering the protein a dominant negative [3], [57]. 16–24 h after transfection, cells were washed once in complete medium, and copper sulfate was added to a final concentration of 0.7 mM to induce gene expression from the metallothionine promoter in *pRmHa3*. Transfection with empty *pRmHa3* was used as the vector control. For immunostaining, S2 cells were fixed in 4% formaldehyde for 20 min, washed with PBS, permeabilized in PBS +0.1% Triton X-100 for 15 min and blocked in PBS +0.1% Triton X-100+1% NGS +1% BSA. Triton X-100 was removed from all washing and blocking solutions for nonpermeabilized staining. Primary antibodies used were guinea pig anti-NijA (1∶500), rabbit anti-myc (1∶500, Abcam), and mouse anti-β-tubulin (1∶500) as permeablization control.

### S2 cell death assay

At different time points after induction (24 h, 40 h or 48 h), cells were resuspended, mixed with 0.4% trypan blue (Gibco) and applied to a hemocytometer for counting. The percentage of dead cells was calculated by dividing the number of trypan blue positive cells by that of the total cells counted. For counting GFP positive cells, different plasmids were co-transfected with *pRmHa3-GFP*. 48 h after the induction, cells were resuspended and 30–50 µl were applied to single wells of a 12-well multi-test slide (MP Biomedicals), allowed to settle for 2 h and then fixed and mounted in Vectashield with DAPI. Pictures were taken with a 20× Plan Aprochromat objective on a Zeiss AxioImager M2 microscope. GFP positive/negative cells were counted from three randomly chosen fields. 600–2500 cells were counted for each sample. For cell death after 100 nM actinomycin D treatment, 5 µg/well dsRNA against NijA was added to 3×10^5^ cells in 250 µl serum-free medium in a 24-well plate. After incubation for 1 h, 500 µl complete medium was added and 48 h later, actinomycin D was added to a final concentration of 100 nM. 6 h after actinomycin D treatment, total numbers of living (trypan blue negative) cells were counted using the hemocytometer and the survival rate was determined relative to the untreated (DMSO), wild-type sample.

## Supporting Information

Supporting Information S1 ***NijA*** **is not required for survival to immune challenge.** Survival of adult males injected with either *M. luteus* (**A**) or *E. coli* (**B**). *NijA* was not required in adults for survival to *M. luteus* or *E. coli*. (**C**) Survival of third instar larvae wounded with a non-sterile fine needle. *NijA* was not required in larvae to survive wounding, as *NijA^D3^* mutants were not significantly different from wild type in their ability to survive wounding (Student's T test). Error bars represent standard error of the mean. Methods: Adult Infection: Adult males were collected from a healthy bottle 24 h after clearing, aged two days in a 25°C incubator, and injected with 69 nl of a log-phase growth culture of either *M. luteus* or *E. coli* using a Nanoject apparatus (Drummond) into the lateral side of the abdomen just below the halteres. 10 animals were placed in a vial containing 10 ml of standard molasses food and allowed to recover at 25°C in a humidified incubator. An adult was scored as dead if it was not standing up, and vials were scored every 24 h. All adults survived the first 5 h. For *M. luteus* three replicates were performed with 20 animals each. For *E. coli* three replicates were performed of 30 animals each. Larval Wounding: Third instar larvae were collected from the food of a healthy bottle and impaled with a fine needle (Fine Science Tools) in the posterior third of the animal near the lateral side to avoid puncturing the gut or damaging the dorsal vessel. 20 larvae were allowed to recover on a grape juice plate with wet yeast in a humidified 25°C incubator. Larvae were scored as dead if they did not respond to gentle prodding with a probe and if the dorsal vessel did not beat. Three replicates were performed of 20 animals each.
(PDF)

Supporting Information S2 ***NijA*** **is not required for Toll-mediated antimicrobial pep- tide induction.** (**A–B**) qPCR analysis of relative *Drosomycin* (*Drs*, A) expression or *Drosocin* (*Dro*, B) expression in male third instar larvae after septic injury with *M. luteus*. *NijA^D3^* homozygotes were able to respond to immune challenge by upregulating both antimicrobial peptides similarly to wild type. (**C**) qPCR analysis of relative *Drosomycin* expression after septic injury, or in *C564>Toll^10b^* larvae where *Tl* is genetically activated in the fat body. *NijA^D3^* homozygous mutants were able to respond to *Tl* gain-of-function in the fat body by increasing *Drosomycin* to levels similar to heterozygous sibling controls. The slight increases in *Drs* and *Dro* observed in *NijA^D3^* untreated larvae in all three panels are not statistically significant. Methods: Larvae were pierced with a fine needle (Fine Science Tools) dipped in a log-phase growth culture of *M. luteus* in LB. qPCR was performed as described in Materials and Methods, except that 2 µl of the cDNA pools were primed with validated primers sets for *rp49* (R^2^<0.99), *Drosocin* (R^2^<0.98), and *Drosomycin* (R^2^<0.99), as previously described by [1]. All values are reported relative to untreated wild-type samples. Each sample was run in triplicate, and a minimum of three independent biological replicates was performed per condition. 1. Leulier F, Lhocine N, Lemaitre B, Meier P (2006) The Drosophila inhibitor of apoptosis protein DIAP2 functions in innate immunity and is essential to resist gram-negative bacterial infection. Mol Cell Biol 26: 7821–7831.
(PDF)

Supporting Information S3 ***NijA*** **is not required for phagocytosis of** ***E. coli*** **.** (**A**) Heat-killed fluorescently labeled *E. coli* particles were injected into third instar larvae, and after 30 min hemocytes were scored *ex vivo* for number of particles engulfed per cell. Cells engulfing five or more particles were considered “super” phagocytosing cells. Both *NijA^D3^* homozygotes and heterozygotes had significantly more super-phagocytosing cells than wild type, indicating that the effect was likely caused by a dominant locus on the *NijA^D3^* chromosome. (**B**) Drosophila S2 cells were incubated with fluorescently labeled *E. coli* and scored using flow cytometry. S2 cells treated with a *NijA-RNAi* construct were able to phagocytose at the same efficiency as wild-type cells. (**C**) Western blot of S2 cell lysates probed with anti-NijA demonstrating a strong reduction in NijA protein in *NijA-RNAi* treated S2 cells. This western was repeated twice. Error bars represent standard error of the mean. Methods: *In vivo* phagocytosis: Wandering 3^rd^ instar larvae from healthy bottles were septically wounded with a fine needle (Fine Science Tools) dipped in a concentrated mix of *E. coli* and *M. luteus*. The larvae were allowed to recover on “drinking plates” at 25°C in a humidified incubator for 2 h. (Drinking plates are grape juice plates with wet yeast, scored with a probe in one quarter of the plate, and the scored areas filled with distilled water.) The larvae were then injected in the lateral side using a Nanoject apparatus (Drummond) with 69 nl of 1.0×10^6^ heat-killed FITC labeled *E. coli* particles (Bioparticles, Molecular Probes/Invitrogen) suspended in phosphate buffered saline (PBS). Larvae were allowed to recover on “drinking plates” for 30 min at 25°C in a humidified incubator. The larval body was torn open in the posterior end with forceps and hemolymph was collected. Hemolymph from five animals was pooled in 10 µl of 0.4% Trypan Blue in PBS to quench fluorescence of the extracellular particles, and all 10 µl loaded on a hemocytometer for scoring. Hemocytes were viewed on the hemocytometer with a Zeiss Imager M2 microscope with a 20× objective, and the number of fluorescent particles per cell was scored. Five or more particles per cell were considered “super” phagocytosers. Each sample was tested in three independent pools of five animals for each genotype. Flow Cytometry Measurements of Phagocytosis: 2.0×10^5^ Drosophila S2 cells were plated in 200 µl complete media in each well of a 24-well plate. Selected wells were treated with 6 µg of dsRNA against *NijA* for 30 mins. All wells were then supplemented with 400 µl of complete media and cultured at 25°C for 48 hrs. The phagocytosis assay was conducted as previously described [1]. Briefly, to each well was added 2 µl of a 1.0×10^6^ particle/µl solution of heat-killed FITC labeled *E. coli* particles (Bioparticles, Molecular Probes/Invitrogen) in PBS. Plates were placed on ice for 30 min then transferred to room temperature for 15 min. Cells were suspended with vigorous pipetting and mixed 1∶1 with 0.4% Trypan Blue to quench extracellular fluorescence. Cells were analyzed on a FACSaria flow cytometry machine (BD Biosciences) for 10,000 events per well, and the phagocytic index (phagocytosis events multiplied by mean fluorescence of phagocytosing cells) was calculated as previously described by Kocks et. al. [2]. Six wells were run on two different days for each condition. 1. Ramet M, Manfruelli P, Pearson A, Mathey-Prevot B, Ezekowitz RA (2002) Functional genomic analysis of phagocytosis and identification of a Drosophila receptor for E. coli. Nature 416: 644–648. 2. Kocks C, Cho JH, Nehme N, Ulvila J, Pearson AM, et al. (2005) Eater, a transmembrane protein mediating phagocytosis of bacterial pathogens in Drosophila. Cell 123: 335–346.
(PDF)

Supporting Information S4 ***NijA*** **is not required for resistance to starvation.** Although *NijA^D3^* homozygotes are significantly more resistant to starvation than wild type, the resistance is likely to be caused by a dominant locus on the *NijA^D3^* chromosome because the *NijA^D3^/+*heterozygote also displays increased resistance to starvation. It appears that *NijA* is not required for resistance to starvation, as the *NijA* homozygote does not have increased resistance compared to the heterozygote. Methods: Adult males were collected from a bottle cleared 24 h prior to collection. Ten males were placed in vials containing two Kimwipes with 1.5 ml sterile water for starvation conditions. Ten males were placed in vials containing 10 ml of standard molasses food for fed conditions. Adult males were scored as dead when they were no longer standing upright. All animals were alive on day two, and all animals were dead by day four in the starvation vials. Three independent replicates were scored concurrently. Error bars represent standard error of the mean.
(PDF)

Supporting Information S5 ***NijA*** **is not required for developmentally programmed cell death in the embryo.** Stage 10 embryos were fixed and stained with anti-tubulin to show embryo morphology and anti-cleaved-caspase 3 to label apoptotic cells. Cleaved-caspase 3 staining in the anterior of the embryo appeared similar in the *NijA^D3^* mutant and the wild-type embryos. Anterior is on the left and dorsal is up. Methods: Embryos from an overnight collection of *w* or homozygous *NijA* mothers were dechorionated in 50% Clorox bleach, fixed at the interface of heptane and 4% formaldehyde (Ted Pella), and deviteillinized in methanol/heptane. Embryos were slowly rehydrated, blocked in 1% Bovine Serum Albumin (BSA) in 1× PBS +0.2% Tween 20 (PBST) for 30 min at room temperature with gentle rocking, and stained overnight at 4°C with rat anti-tubulin at 1∶200 (AbD Serotec, clone YL1/2) and rabbit anti-cleaved-caspase 3 at 1∶50 (Cell Signaling, #9661) diluted in the blocking solution. Embryos were washed several times in PBST, and stained for 2 h at RT with FITC-labeled goat anti-rat and Cy3-labeled goat anti-rabbit, each at 1∶200 in blocking solution. Embryos were washed in PBST, dehydrated with methanol, and mounted in clearing solution (2∶1 Benzyl Benzoate: Benzyl Alcohol). Embryos were photographed using a Zeiss Imager M2 with Apotome. Images are a projection of a Z-series to show all of the caspase-positive cells present in the embryo. All stage 10 embryos of both genotypes were caspase-positive.
(PDF)

Supporting Information S6 **The secreted ectodomain of** ***NijA*** **does not induce cell death.** (**A**) Cells were transfected with *pRmHa3-NijA* (columns 1 and 2) or *pRmHa3-NijA- ectodomain* (columns 3 and 4) and induced for 40 h. To express C-terminal flag-tagged forms, cells were co-transfected with *pRmHa3-GAL4* and *UAS-NijA-flag* (column 5) or *UAS- NijA-ecto-flag* (column 6) and induced for 48 hrs. Percentage of dead cells was determined by counting trypan blue positive cells. (**B**) *NijA* ectodomain was expressed and secreted into the medium. Western blot with anti-flag was performed on cell lysate and medium collected from cells transfected with *pRmHa3-GAL4* and *UAS-NijA-ecto-flag* or *pRmHa3-GAL4* alone (control). (**C**) Localization of *NijA-ecto-flag* shown by immunofluorescence staining with *anti-NijA* (red) and *anti-flag* (green). Cell nuclei were stained in blue by DAPI.
(PDF)
