# Supplementary material for: Drosophila Ninjurin A Induces Nonapoptotic Cell Death
Source: PLoS One. 2012 Sep 28;7(9):e44567. doi: 10.1371/journal.pone.0044567 (PMC3460944; doi:10.1371/journal.pone.0044567)
Supplement: Supporting Information S1 — NijA is not required for survival to immune challenge. Survival of adult males injected with either M. luteus (A) or E. coli (B). NijA was not required in adults for survival to M. luteus or E. coli. (C) Survival of third instar larvae wounded with a non-sterile fine needle. NijA was not required in larvae to survive wounding, as NijAD3 mutants were not significantly different from wild type in their ability to survive wounding (Student's T test). Error bars represent standard error of the mean. Methods: Adult Infection: Adult males were collected from a healthy bottle 24 h after clearing, aged two days in a 25°C incubator, and injected with 69 nl of a log-phase growth culture of either M. luteus or E. coli using a Nanoject apparatus (Drummond) into the lateral side of the abdomen just below the halteres. 10 animals were placed in a vial containing 10 ml of standard molasses food and allowed to recover at 25°C in a humidified incubator. An adult was scored as dead if it was not standing up, and vials were scored every 24 h. All adults survived the first 5 h. For M. luteus three replicates were performed with 20 animals each. For E. coli three replicates were performed of 30 animals each. Larval Wounding: Third instar larvae were collected from the food of a healthy bottle and impaled with a fine needle (Fine Science Tools) in the posterior third of the animal near the lateral side to avoid puncturing the gut or damaging the dorsal vessel. 20 larvae were allowed to recover on a grape juice plate with wet yeast in a humidified 25°C incubator. Larvae were scored as dead if they did not respond to gentle prodding with a probe and if the dorsal vessel did not beat. Three replicates were performed of 20 animals each. (PDF) [file pone.0044567.s001.pdf]

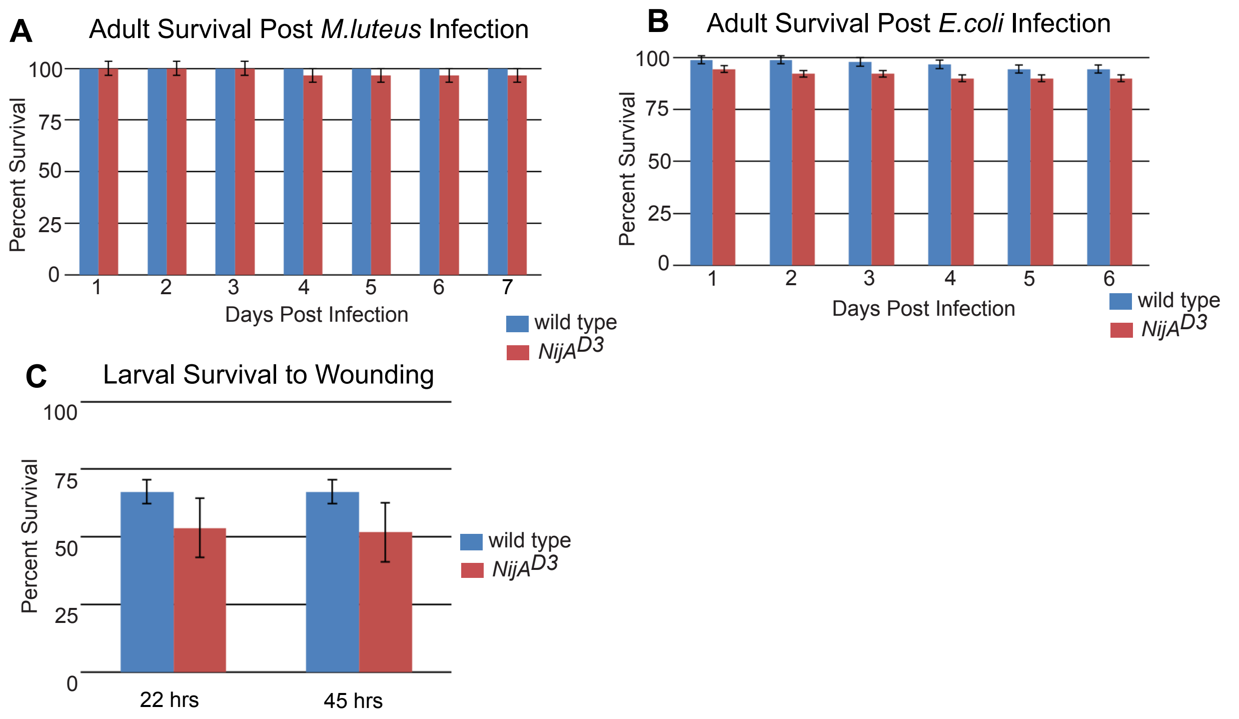

Supporting Information S1. *NijA* is not required for survival to immune challenge. Survival of adult males injected with either *M. luteus* **(A)** or *E. coli* **(B)**. *NijA* was not required in adults for survival to *M. luteus* or *E. coli*. **(C)** Survival of third instar larvae wounded with a non-sterile fine needle. *NijA* was not required in larvae to survive wounding, as $NijA^{D3}$ mutants were not significantly different from wild type in their ability to survive wounding (Student's T test). Error bars represent standard error of the mean.

Methods:

Adult Infection:

Adult males were collected from a healthy bottle 24h after clearing, aged two days in a 25°C incubator, and injected with 69nl of a log-phase growth culture of either *M. luteus* or *E. coli* using a Nanoject apparatus (Drummond) into the lateral side of the abdomen just below the halteres. 10 animals were placed in a vial containing 10ml of standard molasses food and allowed to recover at 25°C in a humidified incubator. An adult was scored as dead if it was not standing up, and vials were scored every 24h. All adults survived the first 5h. For *M. luteus* three replicates were performed with 20 animals each. For *E. coli* three replicates were performed of 30 animals each.

Larval Wounding:

Third instar larvae were collected from the food of a healthy bottle and impaled with a fine needle (Fine Science Tools) in the posterior third of the animal near the lateral side to avoid puncturing the gut or damaging the dorsal vessel. 20 larvae were allowed to recover on a grape juice plate with wet yeast in a humidified 25°C incubator. Larvae were scored as dead if they did not respond to gentle prodding with a probe and if the dorsal vessel did not beat. Three replicates were performed of 20 animals each.
