# Supplementary material for: Drosophila Ninjurin A Induces Nonapoptotic Cell Death
Source: PLoS One. 2012 Sep 28;7(9):e44567. doi: 10.1371/journal.pone.0044567 (PMC3460944; doi:10.1371/journal.pone.0044567)
Supplement: Supporting Information S2 — NijA is not required for Toll-mediated antimicrobial pep- tide induction. (A–B) qPCR analysis of relative Drosomycin (Drs, A) expression or Drosocin (Dro, B) expression in male third instar larvae after septic injury with M. luteus. NijAD3 homozygotes were able to respond to immune challenge by upregulating both antimicrobial peptides similarly to wild type. (C) qPCR analysis of relative Drosomycin expression after septic injury, or in C564>Toll10b larvae where Tl is genetically activated in the fat body. NijAD3 homozygous mutants were able to respond to Tl gain-of-function in the fat body by increasing Drosomycin to levels similar to heterozygous sibling controls. The slight increases in Drs and Dro observed in NijAD3 untreated larvae in all three panels are not statistically significant. Methods: Larvae were pierced with a fine needle (Fine Science Tools) dipped in a log-phase growth culture of M. luteus in LB. qPCR was performed as described in Materials and Methods, except that 2 µl of the cDNA pools were primed with validated primers sets for rp49 (R2<0.99), Drosocin (R2<0.98), and Drosomycin (R2<0.99), as previously described by [1]. All values are reported relative to untreated wild-type samples. Each sample was run in triplicate, and a minimum of three independent biological replicates was performed per condition. 1. Leulier F, Lhocine N, Lemaitre B, Meier P (2006) The Drosophila inhibitor of apoptosis protein DIAP2 functions in innate immunity and is essential to resist gram-negative bacterial infection. Mol Cell Biol 26: 7821–7831. (PDF) [file pone.0044567.s002.pdf]

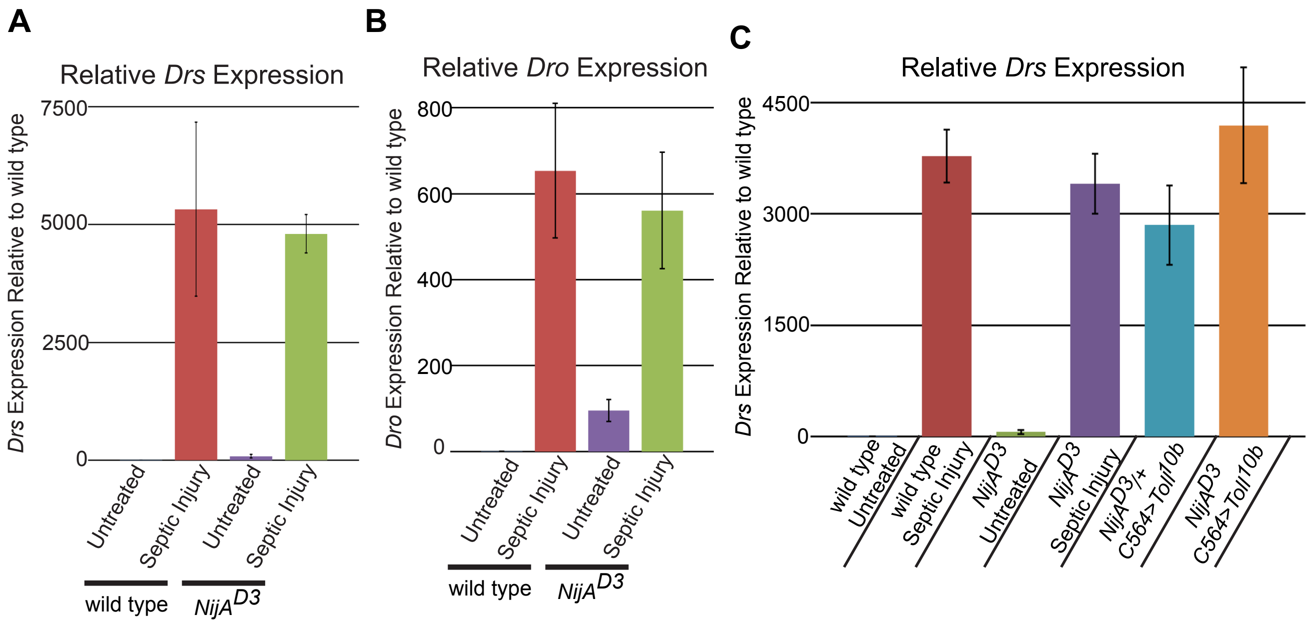

Supporting Information S2. *NijA* is not required for Toll-mediated antimicrobial peptide induction.

(A-B) qPCR analysis of relative *Drosomycin* (*Drs*, A) expression or *Drosocin* (*Dro*, B) expression in male third instar larvae after septic injury with *M. luteus*. *NijA*^{D3} homozygotes were able to respond to immune challenge by upregulating both antimicrobial peptides similarly to wild type. **(C)** qPCR analysis of relative *Drosomycin* expression after septic injury, or in *C564>Toll*^{10b} larvae where *Tl* is genetically activated in the fat body. *NijA*^{D3} homozygous mutants were able to respond to *Tl* gain-of-function in the fat body by increasing *Drosomycin* to levels similar to heterozygous sibling controls. The slight increases in *Drs* and *Dro* observed in *NijA*^{D3}untreated larvae in all three panels are not statistically significant.

Methods:
Larvae were pierced with a fine needle (Fine Science Tools) dipped in a log-phase growth culture of *M. luteus* in LB. qPCR was performed as described in Materials and Methods, except that 2µl of the cDNA pools were primed with validated primers sets for *rp49* (R2<0.99), *Drosocin* (R2<0.98), and *Drosomycin* (R2<0.99), as previously described by [1]. All values are reported relative to untreated wild-type samples. Each sample was run in triplicate, and a minimum of three independent biological replicates was performed per condition.

1. Leulier F, Lhocine N, Lemaitre B, Meier P (2006) The Drosophila inhibitor of apoptosis protein DIAP2 functions in innate immunity and is essential to resist gram-negative bacterial infection. Mol Cell Biol 26: 7821-7831.
