# Supplementary material for: Drosophila Ninjurin A Induces Nonapoptotic Cell Death
Source: PLoS One. 2012 Sep 28;7(9):e44567. doi: 10.1371/journal.pone.0044567 (PMC3460944; doi:10.1371/journal.pone.0044567)
Supplement: Supporting Information S3 — NijA is not required for phagocytosis of E. coli . (A) Heat-killed fluorescently labeled E. coli particles were injected into third instar larvae, and after 30 min hemocytes were scored ex vivo for number of particles engulfed per cell. Cells engulfing five or more particles were considered “super” phagocytosing cells. Both NijAD3 homozygotes and heterozygotes had significantly more super-phagocytosing cells than wild type, indicating that the effect was likely caused by a dominant locus on the NijAD3 chromosome. (B) Drosophila S2 cells were incubated with fluorescently labeled E. coli and scored using flow cytometry. S2 cells treated with a NijA-RNAi construct were able to phagocytose at the same efficiency as wild-type cells. (C) Western blot of S2 cell lysates probed with anti-NijA demonstrating a strong reduction in NijA protein in NijA-RNAi treated S2 cells. This western was repeated twice. Error bars represent standard error of the mean. Methods: In vivo phagocytosis: Wandering 3rd instar larvae from healthy bottles were septically wounded with a fine needle (Fine Science Tools) dipped in a concentrated mix of E. coli and M. luteus. The larvae were allowed to recover on “drinking plates” at 25°C in a humidified incubator for 2 h. (Drinking plates are grape juice plates with wet yeast, scored with a probe in one quarter of the plate, and the scored areas filled with distilled water.) The larvae were then injected in the lateral side using a Nanoject apparatus (Drummond) with 69 nl of 1.0×10^6^ heat-killed FITC labeled E. coli particles (Bioparticles, Molecular Probes/Invitrogen) suspended in phosphate buffered saline (PBS). Larvae were allowed to recover on “drinking plates” for 30 min at 25°C in a humidified incubator. The larval body was torn open in the posterior end with forceps and hemolymph was collected. Hemolymph from five animals was pooled in 10 µl of 0.4% Trypan Blue in PBS to quench fluorescence of the extracellular particles, and all 10 µl loaded on [file pone.0044567.s003.pdf]

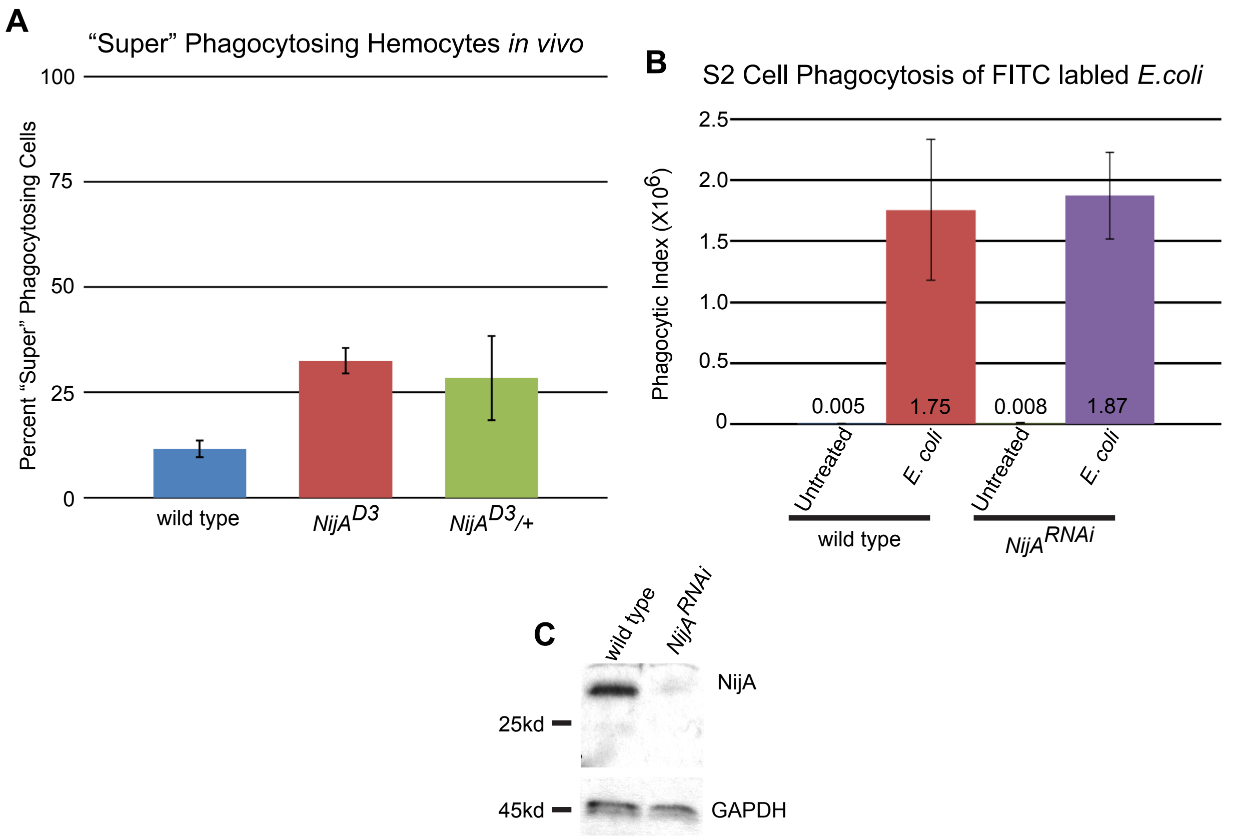

Supporting Information S3. *NijA* is not required for phagocytosis of *E. coli*.
(A) Heat-killed fluorescently labeled *E. coli* particles were injected into third instar larvae, and after 30 min hemocytes were scored *ex vivo* for number of particles engulfed per cell. Cells engulfing five or more particles were considered "super" phagocytosing cells. Both $NijA^{D3}$ homozygotes and heterozygotes had significantly more super-phagocytosing cells than wild type, indicating that the effect was likely caused by a dominant locus on the $NijA^{D3}$ chromosome. **(B)** Drosophila S2 cells were incubated with fluorescently labeled *E. coli* and scored using flow cytometry. S2 cells treated with a *NijA-RNAi* construct were able to phagocytose at the same efficiency as wild-type cells. **(C)** Western blot of S2 cell lysates probed with anti-NijA demonstrating a strong reduction in NijA protein in *NijA-RNAi* treated S2 cells. This western was repeated twice. Error bars represent standard error of the mean.
.

Methods.

In vivo phagocytosis:

Wandering 3rd instar larvae from healthy bottles were septically wounded with a fine needle (Fine Science Tools) dipped in a concentrated mix of *E. coli* and *M. luteus*. The larvae were allowed to recover on “drinking plates” at 25°C in a humidified incubator for 2h. (Drinking plates are grape juice plates with wet yeast, scored with a probe in one quarter of the plate, and the scored areas filled with distilled water.) The larvae were then injected in the lateral side using a Nanoject apparatus (Drummond) with 69nl of $1.0x10^6$ heat-killed FITC labeled *E. coli* particles (Bioparticles, Molecular Probes/Invitrogen) suspended in phosphate buffered saline (PBS). Larvae were allowed to recover on “drinking plates” for 30 min at 25°C in a humidified incubator. The larval body was torn open in the posterior end with forceps and hemolymph was collected. Hemolymph from five animals was pooled in 10μl of 0.4% Trypan Blue in PBS to quench fluorescence of the extracellular particles, and all 10μl loaded on a hemocytometer for scoring. Hemocytes were viewed on the hemocytometer with a Zeiss Imager M2 microscope with a 20X objective, and the number of fluorescent particles per cell was scored. Five or more particles per cell were considered “super” phagocytosers. Each sample was tested in three independent pools of five animals for each genotype.

Flow Cytometry Measurements of Phagocytosis:

$2.0x10^5$ Drosophila S2 cells were plated in 200μl complete media in each well of a 24-well plate. Selected wells were treated with 6μg of dsRNA against *NijA* for 30mins. All wells were then supplemented with 400μl of complete media and cultured at 25°C for 48hrs. The phagocytosis assay was conducted as previously described [1]. Briefly, to each well was added 2μl of a $1.0x10^6$ particle/μl solution of heat-killed FITC labeled *E. coli* particles (Bioparticles, Molecular Probes/Invitrogen) in PBS. Plates were placed on ice for 30 min then transferred to room temperature for 15 min. Cells were suspended with vigorous pipetting and mixed 1:1 with 0.4% Trypan Blue to quench extracellular fluorescence. Cells were analyzed on a FACSaria flow cytometry machine (BD Biosciences) for 10,000 events per well, and the phagocytic index (phagocytosis events multiplied by mean fluorescence of phagocytosing cells) was calculated as previously described by Kocks et. al. [2]. Six wells were run on two different days for each condition.

1. Ramet M, Manfruelli P, Pearson A, Mathey-Prevot B, Ezekowitz RA (2002) Functional genomic analysis of phagocytosis and identification of a Drosophila receptor for E. coli. Nature 416: 644-648.
2. Kocks C, Cho JH, Nehme N, Ulvila J, Pearson AM, et al. (2005) Eater, a transmembrane protein mediating phagocytosis of bacterial pathogens in Drosophila. Cell 123: 335-346.
