# Supplementary material for: Drosophila Ninjurin A Induces Nonapoptotic Cell Death
Source: PLoS One. 2012 Sep 28;7(9):e44567. doi: 10.1371/journal.pone.0044567 (PMC3460944; doi:10.1371/journal.pone.0044567)
Supplement: Supporting Information S4 — NijA is not required for resistance to starvation. Although NijAD3 homozygotes are significantly more resistant to starvation than wild type, the resistance is likely to be caused by a dominant locus on the NijAD3 chromosome because the NijAD3/+heterozygote also displays increased resistance to starvation. It appears that NijA is not required for resistance to starvation, as the NijA homozygote does not have increased resistance compared to the heterozygote. Methods: Adult males were collected from a bottle cleared 24 h prior to collection. Ten males were placed in vials containing two Kimwipes with 1.5 ml sterile water for starvation conditions. Ten males were placed in vials containing 10 ml of standard molasses food for fed conditions. Adult males were scored as dead when they were no longer standing upright. All animals were alive on day two, and all animals were dead by day four in the starvation vials. Three independent replicates were scored concurrently. Error bars represent standard error of the mean. (PDF) [file pone.0044567.s004.pdf]

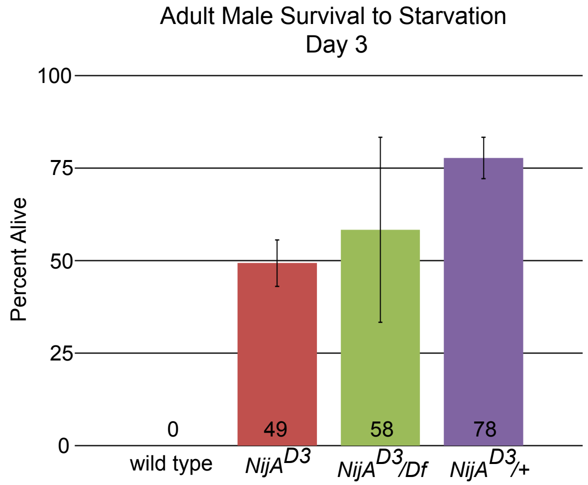

Supporting Information S4. *NijA* is not required for resistance to starvation. Although *NijA*^{D3} homozygotes are significantly more resistant to starvation than wild type, the resistance is likely to be caused by a dominant locus on the *NijA*^{D3} chromosome because the *NijA*$^{D3/+}$ heterozygote also displays increased resistance to starvation. It appears that *NijA* is not required for resistance to starvation, as the *NijA* homozygote does not have increased resistance compared to the heterozygote.

Methods:
Adult males were collected from a bottle cleared 24h prior to collection. Ten males were placed in vials containing two Kimwipes with 1.5ml sterile water for starvation conditions. Ten males were placed in vials containing 10ml of standard molasses food for fed conditions. Adult males were scored as dead when they were no longer standing upright. All animals were alive on day two and all animals were dead by day four in the starvation vials. Three independent replicates were scored concurrently. Error bars represent standard error of the mean.
