# Supplementary material for: Drosophila Ninjurin A Induces Nonapoptotic Cell Death
Source: PLoS One. 2012 Sep 28;7(9):e44567. doi: 10.1371/journal.pone.0044567 (PMC3460944; doi:10.1371/journal.pone.0044567)
Supplement: Supporting Information S5 — NijA is not required for developmentally programmed cell death in the embryo. Stage 10 embryos were fixed and stained with anti-tubulin to show embryo morphology and anti-cleaved-caspase 3 to label apoptotic cells. Cleaved-caspase 3 staining in the anterior of the embryo appeared similar in the NijAD3 mutant and the wild-type embryos. Anterior is on the left and dorsal is up. Methods: Embryos from an overnight collection of w or homozygous NijA mothers were dechorionated in 50% Clorox bleach, fixed at the interface of heptane and 4% formaldehyde (Ted Pella), and deviteillinized in methanol/heptane. Embryos were slowly rehydrated, blocked in 1% Bovine Serum Albumin (BSA) in 1× PBS +0.2% Tween 20 (PBST) for 30 min at room temperature with gentle rocking, and stained overnight at 4°C with rat anti-tubulin at 1∶200 (AbD Serotec, clone YL1/2) and rabbit anti-cleaved-caspase 3 at 1∶50 (Cell Signaling, #9661) diluted in the blocking solution. Embryos were washed several times in PBST, and stained for 2 h at RT with FITC-labeled goat anti-rat and Cy3-labeled goat anti-rabbit, each at 1∶200 in blocking solution. Embryos were washed in PBST, dehydrated with methanol, and mounted in clearing solution (2∶1 Benzyl Benzoate: Benzyl Alcohol). Embryos were photographed using a Zeiss Imager M2 with Apotome. Images are a projection of a Z-series to show all of the caspase-positive cells present in the embryo. All stage 10 embryos of both genotypes were caspase-positive. (PDF) [file pone.0044567.s005.pdf]

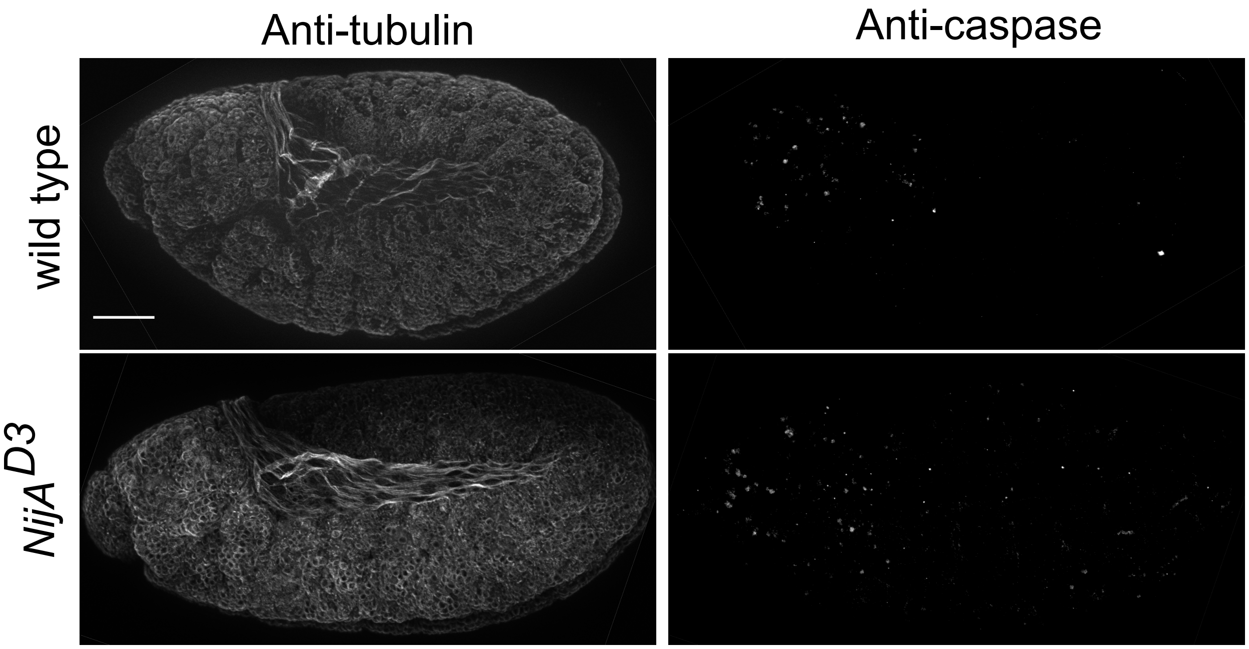

Supporting Information S5. *NijA* is not required for developmentally programmed cell death in the embryo.

Stage 10 embryos were fixed and stained with anti-tubulin to show embryo morphology and anti-cleaved-caspase 3 to label apoptotic cells. Cleaved-caspase 3 staining in the anterior of the embryo appeared similar in the $NijA^{D3}$ mutant and the wild-type embryos. Anterior is on the left and dorsal is up.

Methods.

Embryos from an overnight collection of w^{1118} or homozygous $NijA^{D3}$ mothers were dechorionated in 50% Clorox bleach, fixed at the interface of heptane and 4% formaldehyde (Ted Pella), and deviteillinized in methanol/heptane. Embryos were slowly rehydrated, blocked in 1% Bovine Serum Albumin (BSA) in 1X PBS + 0.2% Tween 20 (PBST) for 30 min at room temperature with gentle rocking, and stained overnight at 4°C with rat anti-tubulin at 1:200 (AbD Serotec, clone YL1/2) and rabbit anti-cleaved-caspase 3 at 1:50 (Cell Signaling, #9661) diluted in the blocking solution. Embryos were washed several times in PBST, and stained for 2h at RT with FITC-labeled goat anti-rat and Cy3-labeled goat anti-rabbit, each at 1:200 in blocking solution. Embryos were washed in PBST, dehydrated with methanol, and mounted in clearing solution (2:1 Benzyl Benzoate: Benzyl Alcohol). Embryos were photographed using a Zeiss Imager M2 with Apotome. Images are a projection of a Z-series to show all of the caspase-positive cells present in the embryo. All stage 10 embryos of both genotypes were caspase-positive.
