# Supplementary material for: Drosophila Ninjurin A Induces Nonapoptotic Cell Death
Source: PLoS One. 2012 Sep 28;7(9):e44567. doi: 10.1371/journal.pone.0044567 (PMC3460944; doi:10.1371/journal.pone.0044567)
Supplement: Supporting Information S6 — The secreted ectodomain of NijA does not induce cell death. (A) Cells were transfected with pRmHa3-NijA (columns 1 and 2) or pRmHa3-NijA- ectodomain (columns 3 and 4) and induced for 40 h. To express C-terminal flag-tagged forms, cells were co-transfected with pRmHa3-GAL4 and UAS-NijA-flag (column 5) or UAS- NijA-ecto-flag (column 6) and induced for 48 hrs. Percentage of dead cells was determined by counting trypan blue positive cells. (B) NijA ectodomain was expressed and secreted into the medium. Western blot with anti-flag was performed on cell lysate and medium collected from cells transfected with pRmHa3-GAL4 and UAS-NijA-ecto-flag or pRmHa3-GAL4 alone (control). (C) Localization of NijA-ecto-flag shown by immunofluorescence staining with anti-NijA (red) and anti-flag (green). Cell nuclei were stained in blue by DAPI. (PDF) [file pone.0044567.s006.pdf]

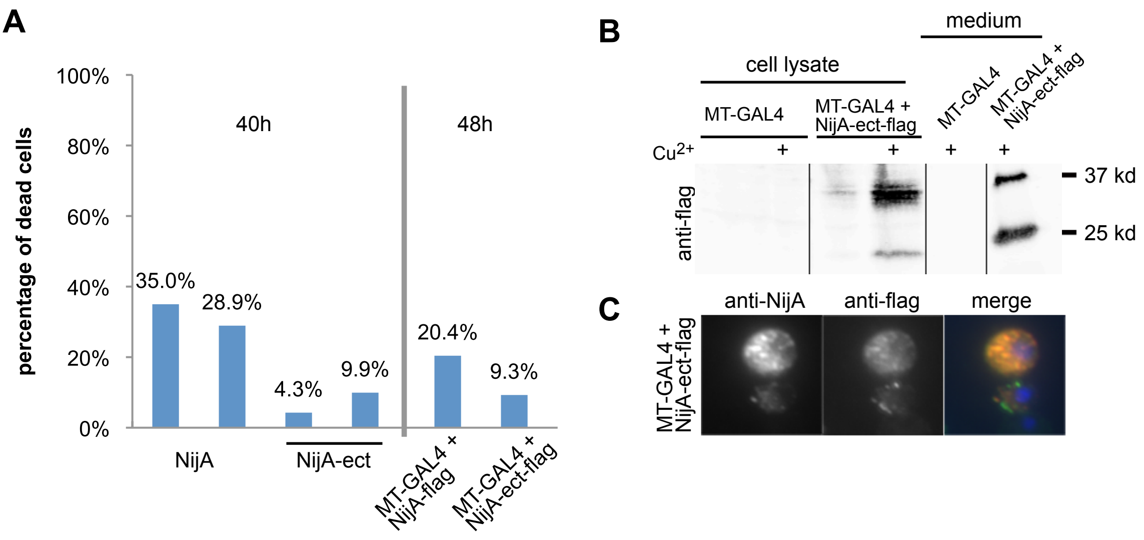

Supporting Information S6. The secreted ectodomain of *NijA* does not induce cell death.

(A) Cells were transfected with *pRmHa3-NijA* (columns 1 and 2) or *pRmHa3-NijA-ectodomain* (columns 3 and 4) and induced for 40h. To express C-terminal flag-tagged forms, cells were co-transfected with *pRmHa3-GAL4* and *UAS-NijA-flag* (column 5) or *UAS-NijA-ecto-flag* (column 6) and induced for 48 hrs. Percentage of dead cells was determined by counting trypan blue positive cells. **(B)** *NijA* ectodomain was expressed and secreted into the medium. Western blot with *anti-flag* was performed on cell lysate and medium collected from cells transfected with *pRmHa3-GAL4* and *UAS-NijA-ecto-flag* or *pRmHa3-GAL4* alone (control). **(C)** Localization of *NijA-ecto-flag* shown by immunofluorescence staining with *anti-NijA* (red) and *anti-flag* (green). Cell nuclei were stained in blue by DAPI.
